# Multicenter CT phantoms public dataset for radiomics reproducibility tests

**DOI:** 10.1002/mp.13385

**Published:** 2019-01-29

**Authors:** Petros Kalendralis, Alberto Traverso, Zhenwei Shi, Ivan Zhovannik, René Monshouwer, Martijn P. A. Starmans, Stefan Klein, Elisabeth Pfaehler, Ronald Boellaard, Andre Dekker, Leonard Wee

**Affiliations:** ^1^ MAASTRO Clinic and School for Oncology and Development Biology (GROW) Maastricht University Medical Centre+ 6229 ET Maastricht The Netherlands; ^2^ Department of Radiation Oncology Radboud University Medical Center 6525 GC Nijmegen The Netherlands; ^3^ Department of Radiology and Nuclear Medicine Erasmus Medical Centre 3015 GD Rotterdam The Netherlands; ^4^ Department of Medical Informatics Erasmus Medical Centre 3015 GD Rotterdam The Netherlands; ^5^ University Medical Center Groningen 9713 GZ Groningen The Netherlands; ^6^ Department of Radiology and Nuclear Medicine VU Medical Center 1081 HV Amsterdam The Netherlands

## Abstract

**Purpose:**

The aim of this paper is to describe a public, open‐access, computed tomography (CT) phantom image set acquired at three centers and collected especially for radiomics reproducibility research. The dataset is useful to test radiomic features reproducibility with respect to various parameters, such as acquisition settings, scanners, and reconstruction algorithms.

**Acquisition and validation methods:**

Three phantoms were scanned in three independent institutions. Images of the following phantoms were acquired: Catphan 700 and COPDGene Phantom II (Phantom Laboratory, Greenwich, NY, USA), and the Triple modality 3D Abdominal Phantom (CIRS, Norfolk, VA, USA). Data were collected at three Dutch medical centers: MAASTRO Clinic (Maastricht, NL), Radboud University Medical Center (Nijmegen, NL), and University Medical Center Groningen (Groningen, NL) with scanners from two different manufacturers Siemens Healthcare and Philips Healthcare. The following acquisition parameter were varied in the phantom scans: slice thickness, reconstruction kernels, and tube current.

**Data format and usage notes:**

We made the dataset publically available on the Dutch instance of “Extensible Neuroimaging Archive Toolkit‐XNAT” (https://xnat.bmia.nl). The dataset is freely available and reusable with attribution (Creative Commons 3.0 license).

**Potential applications:**

Our goal was to provide a findable, open‐access, annotated, and reusable CT phantom dataset for radiomics reproducibility studies. Reproducibility testing and harmonization are fundamental requirements for wide generalizability of radiomics‐based clinical prediction models. It is highly desirable to include only reproducible features into models, to be more assured of external validity across hitherto unseen contexts. In this view, phantom data from different centers represent a valuable source of information to exclude CT radiomic features that may already be unstable with respect to simplified structures and tightly controlled scan settings. The intended extension of our shared dataset is to include other modalities and phantoms with more realistic lesion simulations.

## 1 Introduction

Computer‐aided analysis of clinical radiological images offers a data‐at‐large‐scale approach toward personalized medicine^1^ wherein tumor phenotype may be inferred using images of the entire tumor instead of selective sample biopsies. On the premise that phenotypic variability affects clinical outcome,^2^ medical imaging offers an efficient and noninvasive method to determine prognosis.

This approach has immense potential to support clinical decision‐making in the personalized medicine paradigm,^3^ that is, which would be a superior choice of treatment for a given person. Studies in the active field of image‐derived markers (i.e., “radiomics”) strongly suggest that tomographic images do indeed embed more prognostic information than may be seen by an unassisted human eye.^4^, ^5^, ^6^, ^7^, ^8^ In order to be widely generalizable and have meaningful clinical use, it is essential that reproducibility of features can be tested in phantoms,^9^, ^10^ in addition to validating models in human subjects across different settings and multiple independent institutions.^11^, ^12^, ^13^

Studies have shown that feature reproducibility may be affected by differences in image acquisition parameters, such as slice thickness and reconstruction algorithm.^14^, ^15^, ^16^, ^17^ Since clinical image acquisition protocols are one of the major sources of variation among different hospitals, phantoms allow testing, comparison, and harmonization of radiomic features in similar vein to diagnostic imaging quality assurance. We hypothesize that even simplified phantoms allow us to test for radiomic features that may already become unstable even under tightly constrained conditions.

In this data publication, we offer computed tomography (CT) scans of simple phantoms across three Dutch academic medical centers for open access. We chose to start with CT since this modality is readily available in many centers and is a workhorse imaging modality for radiotherapy intervention planning. In many clinics, CT scanners are mature technology with well‐established protocols for calibration, quality assurance, and routine maintenance.

## 2 Acquisition and validation methods

### 2.A. Phantoms

#### 2.A.1. Catphan 700

To obtain a baseline for overall CT scanner performance, we scanned a Catphan 700 phantom (Phantom Laboratory, Greenwich, NY, USA) that had been designed specifically for routine quality assurance on CT scanners. It is only suitable for use in CT, and contains test modules for contrast, geometric accuracy, and spatial resolution.^18^, ^19^

#### 2.A.2. COPDGene Phantom II

The COPDGene Phantom II (Phantom Laboratory, Greenwich, NY, USA) was designed for thoracic CT quality assurance in prospective clinical trials (specifically asthma and chronic obstructive pulmonary disorder) with guidance from the Quantitative Image Biomarker Alliance Technical Committee. We used the CCT162 version, which included the standard version CTP698 with two additional supports and acrylic end‐plates for stabilization of the phantom during the scanning. An outer polyurethane ring simulated tissue attenuation while an internal oval body (15 cm × 25 cm) simulated lung attenuation. The inner oval held a number of cylindrical cavities for foam, acrylic, and water,^20^, ^21^ as well as a number of internal structures simulating different‐sized bronchi.

#### 2.A.3. Triple modality 3D Abdominal Phantom

A 3D multimodality Abdominal Phantom (CIRS, Norfolk, Virginia, USA) measuring 26 cm × 12.5 cm × 19 cm^22^ was designed to be used for liver biopsy training under guidance by CT, magnetic resonance imaging, or ultrasonography. We scanned Model 057A that simulated the abdomen of a small adult. The materials encased within the phantom represented the liver, portal vein, kidneys, bottom of the lungs, abdominal aorta, vena cava, lumbar spine, and six lowest ribs.

### 2.B. Image acquisition

The images used in our study were acquired using three different CT scanners at independent Dutch centers: MAASTRO Clinic (Maastricht), Radboud University Medical Center (Nijmegen) and University Medical Center Groningen (Groningen). The standard clinical operating procedures for thoracic and abdominal radiotherapy planning CT scans at each of the three centers were used to generate a baseline scan of each phantom. These baseline parameters are stated in Tables 1 and 2, for the Phantom Laboratory and CIRS phantoms, respectively.

**Table 1:** CT scanner details and image acquisition parameters for baseline scans of the Catphan 700 and COPDGene Phantom II in each of the participating clinics

Parameters	DICOM tags	*MAASTRO Clinic (MAAS)*	Radboud University Medical Center (RADB)	University Medical Center Groningen (UMCG)
Catphan 700/COPDGene Phantom II baseline scan parameters				
Manufacturer	(0008, 0070)	Siemens	Phillips	Siemens
Model	(0008, 1090)	Biograph 40	Brilliance Big Bore	Biograph 64
Software Version	(0018, 1020)	syngo CT 2006A	3.6.6	VG60A
Slice thickness (mm)	(0018, 0050)	3	3	3
TUBE VOLTAGE (KV)	(0018, 0060)	120	120	80
Reconstruction diameter (mm)	(0018, 1100)	500	255	239
Tube current (mA)	(0018, 1151)	39	134	149
EXPOSURE (mAs)	(0018, 1152)	24	124	53
Convolution kernel	(0018, 1210)	B31f	B	I30f
Rows	(0028, 0010)	512	1024	512
Columns	(0028, 0011)	512	1024	512
Pixel spacing	(0028, 0030)	0.98	0.25	0.46
Bits stored	(0028, 0101)	12	12	12
High bit	(0028, 0102)	11	11	11
Rescale offset	(0028, 1052)	−1024	−1024	−1024
Rescale slope	(0028, 1053)	1	1	1

**Table 2:** CT scanner details and image acquisition parameters for baseline scans of the multimodality CIRS Abdominal Phantom in each of the participating clinics

Parameters	DICOM tags	MAASTRO Clinic (MAAS)	Radboud University Medical Center (RADB)	University Medical Center Groningen (UMCG)
Triple modality 3D Abdominal Phantom baseline scan parameters				
Manufacturer	(0008, 0070)	Siemens	Phillips	Siemens
Model	(0008, 1090)	Biograph 40	Brilliance Big Bore	Biograph 64
Software Version	(0018,1020)	syngo CT 2006A	3.6.6	VG60A
Manufacturer	(0008, 0070)	Siemens	Phillips	Siemens
TUBE VOLTAGE (KV)	(0018, 0060)	120	120	80
Reconstruction diameter (mm)	(0018, 1100)	500	255	239
Tube current (mA)	(0018, 1151)	118	190	18
EXPOSURE (mAs)	(0018, 1152)	73	175	9
Convolution kernel	(0018, 1210)	B30f	B	I30f
Rows	(0028, 0010)	512	512	512
Columns	(0028, 0011)	512	512	512
Pixel spacing	(0028, 0030)	0.98	0.75	0.59
Bits stored	(0028, 0101)	12	12	12
High bit	(0028, 0102)	11	11	11
Rescale offset	(0028, 1052)	−1024	−1024	−1024
Rescale slope	(0028, 1053)	1	1	1

We subsequently applied perturbations to imaging settings of the baseline scan. We adjusted the following parameters strictly one at a time and saved each scan: slice thickness (1, 3, and 5 mm), reconstruction kernels (between three and five settings depending on the scanner), and current‐exposure product (50, 150, and 300 mAs). The individual setting for each scan is given in Tables 3 and 4, for the Phantom Laboratory and CIRS phantoms, respectively.

**Table 3:** The individual scan settings for the Catphan 700 and COPD II phantoms from the participating different Dutch clinics

Subject	Institution	Slice thickness (mm)	Voltage (kvp)	Current (mA)	Exposure (mAs)	Convolution kernel
Collection: series 1 — Catphan 700 and COPD II individual subject scan settings						
CatPhan‐01‐MAAS	MAASTRO	3	120	39	24	B31f
CatPhan‐01‐RADB	Radboud	3	120	134	124	B
CatPhan‐01‐UMCG	Groningen	3	80	165.5	58.5	I30f
COPD‐001‐MAAS	MAASTRO	3	120	130	80.5	B31f
COPD‐001‐RADB	Radboud	3	120	210	194	B
COPD‐001‐UMCG	Groningen	3	120	191	68	I30f
COPD‐002‐MAAS	MAASTRO	1	120	112.5	69.5	B31f
COPD‐002‐RADB	Radboud	1	120	210	194	B
COPD‐002‐UMCG	Groningen	1	120	205	73	I30f
COPD‐003‐MAAS	MAASTRO	5	120	106.5	66	B31f
COPD‐003‐RADB	Radboud	5	120	210	194	B
COPD‐003‐UMCG	Groningen	5	120	195	69	I30f
COPD‐004‐MAAS	MAASTRO	3	120	91	56	B31f
COPD‐004‐RADB	Radboud	3	120	54	50	B
COPD‐004‐UMCG	Groningen	3	120	140	50	I30f
COPD‐005‐MAAS	MAASTRO	3	120	80	50	B31f
COPD‐005‐RADB	Radboud	3	120	108	100	B
COPD‐005‐UMCG	Groningen	3	120	280	100	I30f
COPD‐006‐MAAS	MAASTRO	3	120	130	80.5	B41f
COPD‐006‐RADB	Radboud	3	120	325	300	B
COPD‐006‐UMCG	Groningen	3	120	660	300	I30f
COPD‐007‐MAAS	MAASTRO	3	120	130	80.5	B41f
COPD‐007‐RADB	Radboud	3	120	210	194	A
COPD‐007‐UMCG	Groningen	3	100	230	104	I40f
COPD‐008‐MAAS	MAASTRO	3	120	130	80.5	B75f
COPD‐008‐RADB	Radboud	3	120	210	194	C
COPD‐008‐UMCG	Groningen	3	100	231	104	I44f
COPD‐009‐MAAS	MAASTRO	3	120	130	80.5	B60f
COPD‐009‐RADB	Radboud	3	120	210	194	E
COPD‐009‐UMCG	Groningen	3	100	236	107	I49f
COPD‐010‐MAAS	MAASTRO	3	120	130	80.5	B80f
COPD‐010‐RADB	Radboud	3	120	210	194	L
COPD‐010‐UMCG	Groningen	3	100	232	105	I50f
COPD‐011‐UMCG	Groningen	3	100	238	108	I70f
COPD‐012‐UMCG	Groningen	3	100	236	107	B30f

**Table 4:** The individual settings of the Triple modality 3D Abdominal Phantoms from the three participating Dutch clinics

Subject	Institution	Slice thickness (mm)	Voltage (kvp)	Current (mA)	Exposure (mAs)	Convolution kernel
Collection: series 2 — CIRS multimodality phantom individual subject scan settings						
CIRS‐AB‐001‐MAAS	MAASTRO	3	120	118	73	B30f
CIRS‐AB‐001‐RADB	Radboud	3	120	190	175	B
CIRS‐AB‐001‐UMCG	Groningen	3	100	100	50	I30f
CIRS‐AB‐002‐MAAS	MAASTRO	1	120	133	83	B30f
CIRS‐AB‐002‐RADB	Radboud	1	120	190	175	B
CIRS‐AB‐002‐UMCG	Groningen	1	100	95	47	I30f
CIRS‐AB‐003‐MAAS	MAASTRO	5	120	136	85	B30f
CIRS‐AB‐003‐RADB	Radboud	5	120	190	175	B
CIRS‐AB‐003‐UMCG	Groningen	5	100	98	49	I30f
CIRS‐AB‐004a‐UMCG	Groningen	3	120	100	50	I30f
CIRS‐AB‐004b‐UMCG	Groningen	3	120	100	50	I30f
CIRS‐AB‐004‐MAAS	MAASTRO	3	120	141	88	B30f
CIRS‐AB‐004‐RADB	Radboud	3	120	54	50	B
CIRS‐AB‐005a‐UMCG	Groningen	3	120	200	100	I30f
CIRS‐AB‐005b‐UMCG	Groningen	3	120	200	100	I30f
CIRS‐AB‐005‐MAAS	MAASTRO	1	120	137	85	B30f
CIRS‐AB‐005‐RADB	Radboud	3	120	108	100	B
CIRS‐AB‐006‐MAAS	MAASTRO	5	120	137.5	85.5	B30f
CIRS‐AB‐006‐RADB	Radboud	3	120	325	300	B
CIRS‐AB‐006‐UMCG	Groningen	3	120	600	300	I30f
CIRS‐AB‐007‐RADB	Radboud	3	120	190	175	A
CIRS‐AB‐007‐UMCG	Groningen	3	100	98	49	I40f
CIRS‐AB‐008‐RADB	Radboud	3	120	190	175	C
CIRS‐AB‐008‐UMCG	Groningen	3	100	98	49	I44f
CIRS‐AB‐009‐RADB	Radboud	3	120	190	175	D
CIRS‐AB‐009‐UMCG	Groningen	3	100	96	48	I49f
CIRS‐AB‐010‐UMCG	Groningen	3	100	97	48	I50f
CIRS‐AB‐011‐UMCG	Groningen	3	100	98	49	I70f
CIRS‐AB‐012‐UMCG	Groningen	3	100	97	48	B30f

### 2.C. Image annotations

CatPhan 700 images were only used for image quality assessment of the baseline scans between participating centers, therefore, no annotations were added to the scans.

**Figure 1:**
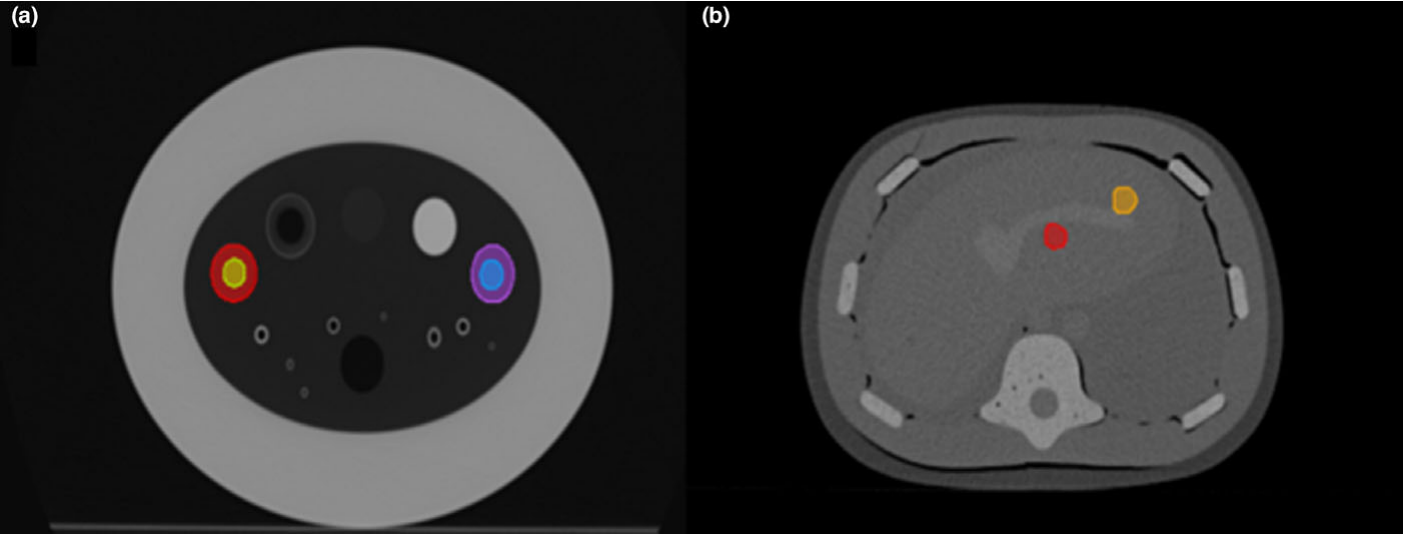
The delineated spherical ROIs within two of the inserts cavities for the COPD and Triple modality 3D Abdominal Phantoms are presented in (a) and (b), respectively.

Regions of interest (ROIs) on the COPDGene and Abdominal Phantoms were manually delineated in MIRADA DBx (version 1.2.0.59, Mirada Medical, Oxford, United Kingdom). In the COPD phantom, we delineated four distinct spherical ROIs within two of the insert cavities. In the multimodality phantom, we delineated two different ROIs corresponding to two of the simulated liver lesions, one large and one small (as shown in Fig. 1). The delineations were performed by one medical physicist at MAASTRO Clinic. All images and annotations were then exported as Digital Imaging and Communications in Medicine (DICOM)‐Radiotherapy (RT) objects.

### 2.D. Data format and usage notes

Our scans are made open access via an instance of the Extensible Neuroimaging Archive Toolkit (XNAT) hosted within Dutch national research infrastructure (TraIT, http://www.ctmm-trait.nl).^23^ XNAT is an open source platform for imaging‐based research and clinical investigations, which manages access to different datasets compartmentalized into separate projects (i.e., collections). Within each collection, XNAT permits browsing of individual cases. The platform supports direct uploading of DICOM images and DICOM‐RT objects (plan, structure set, and dose grid) with *http* file transfer.^24^ Studies in XNAT can be queried and retrieved by means of an API (Application Programming Interface) in the Python programming language by installing the *xnat* library (https://pypi.org/project/xnat/).

The Phantom Laboratory COPD phantom images have been uploaded to the XNAT collection STWSTRATEGY‐Phantom_Series1: (https://xnat.bmia.nl/data/projects/stwstrategyps1).

The CIRS multimodality Abdominal Phantom images have been uploaded to the XNAT collection STWSTRATEGY‐Phantom_Series2: (https://xnat.bmia.nl/data/projects/stwstrategyps2).

The Phantom Laboratories Catphan 700 phantom images have been uploaded to the XNAT collection STW‐STRATEGY‐Phantom_Series3: (https://xnat.bmia.nl/data/projects/stwstrategyps3).

In each of the above collections, the subject identifier matches exactly the names shown in the leftmost column of Tables 3 and 4. DICOM‐formatted images and the annotations as DICOM‐RTStruct objects are nested under the subject level. A python script for downloading an entire collection is available here: (https://github.com/maastroclinic/XNAT-collections-download-script).

The images of the Catphan 700 quality assurance phantom from each center were analyzed online on the quality assurance tests webpage of the ImageOwl company (https://catphanqa.imageowl.com/). Clinical lung CT imaging protocols were used as the reference baseline for radiomics studies, rather than the vendors’ service scan setting. The ImageOWL vendor service provides a detailed quality assurance analysis along with the implementation of linearity and sensitometry plots, noise measurements, and the spatial linearity. All quality assurance test parameters were within tolerance for the clinical lung scan settings used as the reference. The quality assurance reports can be found in Data S1.

## 3 Discussion

We have made publically available multicenter phantom CT scans to support investigations in radiomics repeatability and reproducibility, specifically to identify features that may be unstable with respect to image acquisition settings in simplified geometry.

Radiomics reproducibility may be investigated as a function of: scanner manufacturer/scanner type, slice thickness, tube current (i.e., signal to noise ratio), and reconstruction algorithms. We invite the radiomics community to make use of our dataset for research by extracting radiomic features with their own processing pipelines and comparing the results with other investigators. We also invite the community to contact us in order to share the results of their computations. For the next steps, we intend to host the computed features set from the open source library *pyradiomics v2* (https://github.com/Radiomics/pyradiomics)^25^ as well as the associated DICOM image metadata on a public open‐access website (http://www.radiomics.org).

This is a fundamental step toward improving benchmarking and standardization of the radiomics field of study. This is in support of valuable harmonization projects such as the IBSI (Image Biomarker Standardization Initiative).^26^ The features and metadata will be made available as linked Resource Descriptor Format (RDF) objects labeled with a dedicated radiomic‐specific semantic web ontology (https://bioportal.bioontology.org/ontologies/RO), such that the data can be queried through the SPARQL language. To assist the radiomics community with data sharing, a standard tabular template and conversion script to RDF will also be provided at http://www.radiomics.org.

A number of key limitations in the data must be noted at the present time. First, as explicitly declared by the phantom manufacturers, the phantoms used in this study had not been designed with the specific aim of simulating standard radiomic features. It is presently not fully understood exactly what should be used as a canonical set of imaging features.

Secondly, we posit that the so‐called “test lesions” within the current phantoms represent oversimplified geometries and relatively uniformly dense material. Complex texture patterns and shape features are not well represented in such simple phantoms. However, these phantoms do present a preliminary opportunity for investigating reproducibility of radiomic features, thus we may be able to test for certain features that already unstable in simplified conditions. We would assert that a feature that is not reproducible in such a constrained setting might be unlikely to be highly reproducible in multi‐institutional human studies. To improve on the current situation, the dataset might be expanded by scans of more phantoms that contain more realistic tumor‐mimicking inserts. These may prove to be more suitable for selecting stable features for inclusion in radiomic investigations.

One example of a public phantom dataset which is available on “The Cancer Imaging Archive‐TCIA” is the Credence Cartridge Radiomics (CCR) Phantom (https://wiki.cancerimagingarchive.net/display/Public/Credence+Cartridge+Radiomics+Phantom+CT+Scans). The CCR phantom collection has a similar goal as our study, the investigation of the reproducibility of radiomic features. There is a significant factor that differentiates the CCR phantom public dataset from our phantoms public collections. The structure of the CCR phantom which includes ten cartridges, each with a unique texture, addresses only the question of repeatability and reproducibility of textural features.

Lastly, while we have started with CT as the most commonly available imaging modality in our field, we intend to expand this collection to include positron emission tomography (PET) and magnetic resonance imaging (MRI).

In addition to making available multicenter and multimodality phantoms for radiomics reproducibility studies, future work in this field should make publicly accessible DICOM metadata and image preprocessing steps, so as to make radiomics studies as findable, accessible, interoperable, reusable (FAIR) as possible. To this end, image metadata needs to be linked to the features using publicly available Semantic DICOM (SEDI) ontology^27^ and the Radiomics ontology needs to extended to cover image preprocessing.

## 4 Conclusion

We offer a publicly accessible multicenter CT phantom dataset with carefully controlled image acquisition parameters to support reproducibility research in the field of radiomics. The dataset is hosted in a well‐established and publicly funded XNAT instance. The data are shared under a Creative Commons Attribution 3.0 License (free to browse, download, and use at no cost for scientific and educational purposes). The dataset is offered to the radiomics community to compare simple features extracted with different software pipelines as well as to identify features that may not be stable with respect to image acquisition conditions even under highly simplified conditions. Our unique contribution to the field is to investigate the robustness of each radiomic feature with respect to different scanning acquisition parameters.

## Conflict of interest

The authors declare no conflict of interests pertaining to the above scientific work.

## Supporting information


**Data S1:** Supplementary material with all the information used for the analysis of the scans of the quality assurance phantom Catphan 700.
