# Supplementary material for: Multicenter CT phantoms public dataset for radiomics reproducibility tests
Source: Med Phys. 2019 Jan 29;46(3):1512–8. doi: 10.1002/mp.13385 (PMC6849778; doi:10.1002/mp.13385)
Supplement: Supplementary file 1 — Data S1: Supplementary material with all the information used for the analysis of the scans of the quality assurance phantom Catphan 700. [file MP-46-1512-s001.pdf]

imageOwl Catphan® QA

Petros Kalendralis |

View report

Maastro Clinic 2018-06-06 09:04:15

SIEMENS Biograph 40 1011 / Petros Kalendralis

Sensitometry

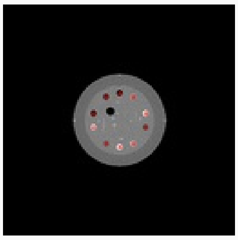

File name: **CT.1.3.12.2.1107.5.1.4.1011.30000018020608084628100001105**, series description: **Thorax 3.0 B31f**, module ID: **CTP682**, Total collimation width: **N/A mm**, kVp: **120**, mA: **39**, time: **500 ms**
slice thickness: **3.00 mm**, recon FOV: **500 mm**, recon filter: **B31f**, scan mode: **N/A**

Linearity plot

CT number vs attenuation coefficient

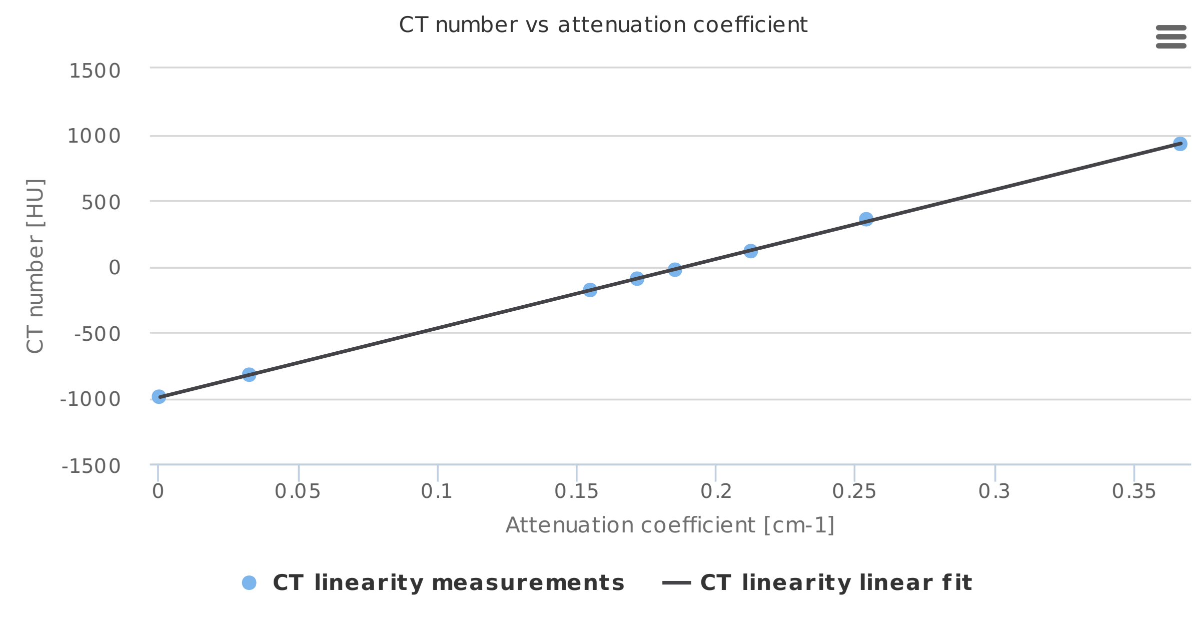

Air [HU]

Measured CT number of air

-992

Lung foam [HU]

Measured CT number of lung foam

-825

PMP [HU]
Measured CT number of PMP

-182

LDPE [HU]
Measured CT number of LDPE

-96

Polystyrene [HU]
Measured CT number of polystyrene

-28

Acrylic [HU]
Measured CT number of acrylic

113

Hydroxyapatite 20% [HU]
Measured CT number of 20% hydroxyapatite

230

Delrin [HU]
Measured CT number of delrin

355

Hydroxyapatite 50% [HU]
Measured CT number of 50% hydroxyapatite

648

Teflon [HU]
Measured CT number of teflon

927

Contrast scale [$cm^{-1}HU^{-1}$]
Contrast scale, slope of linearity plot

0.00019

Effective energy
Effective energy calculated by fitting to table values for keV and attenuation coeffcients

73

Residual plot
Total residuals for fitting table keV values to measured values

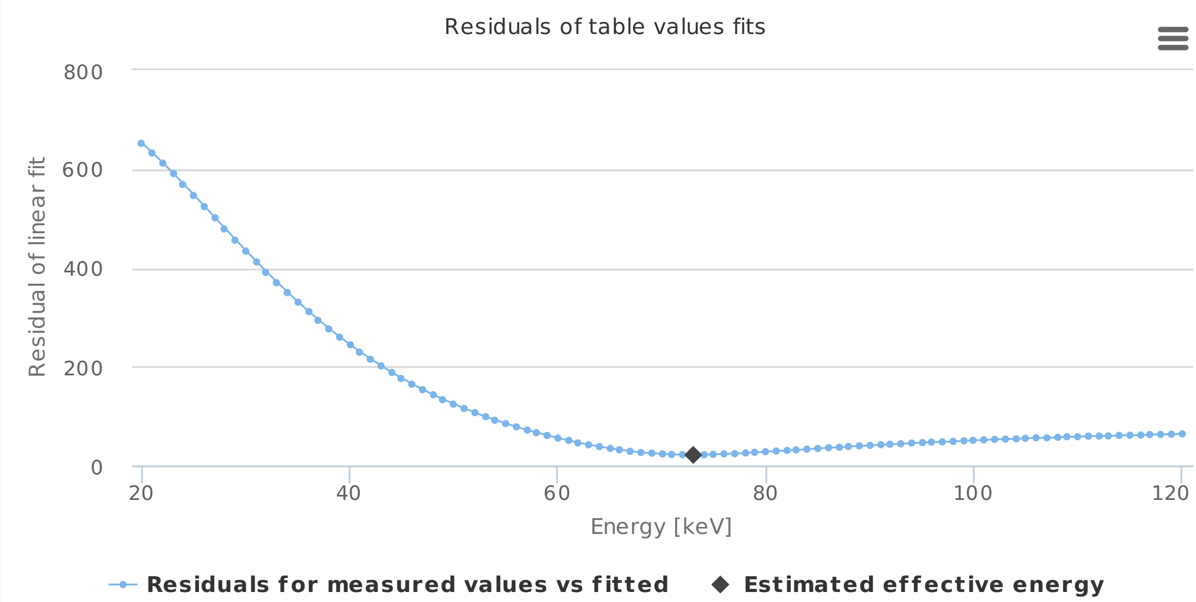

Sensitometry plot

CT numbers vs estimated keV from table for each material

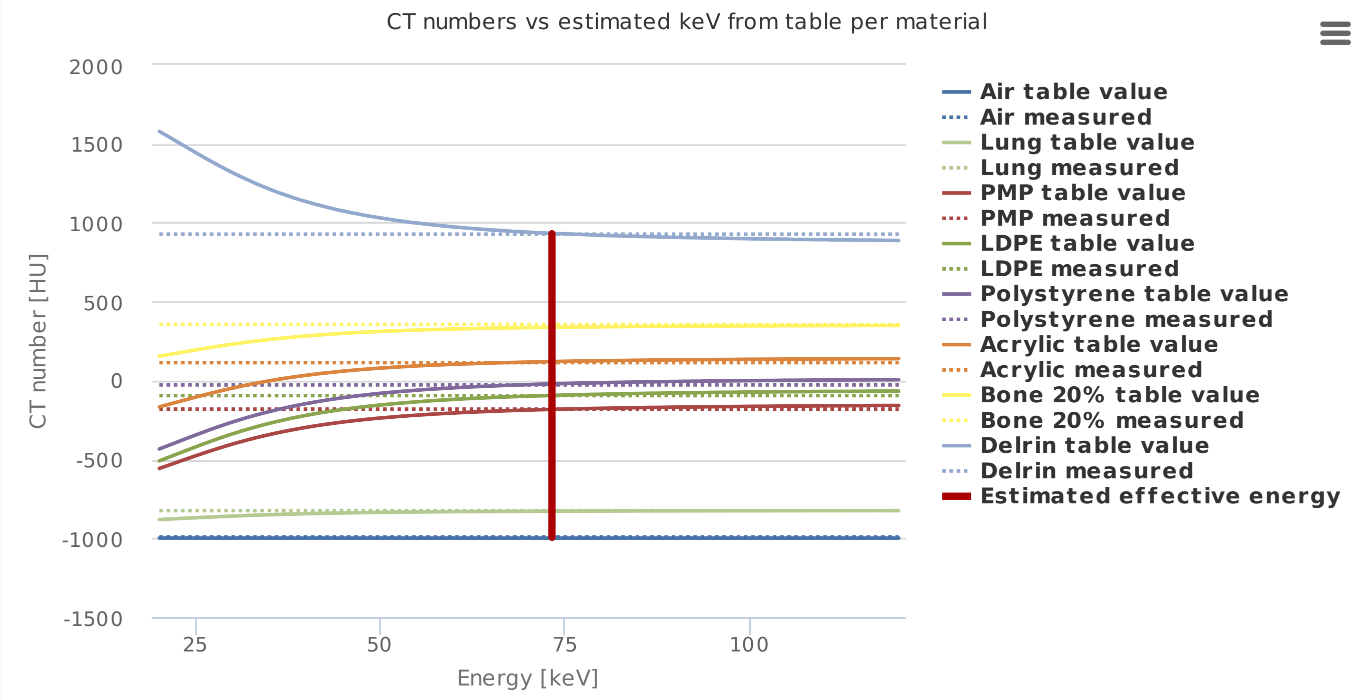

Spatial linearity

Measured pixel spacing [mm]

Measured pixel spacing

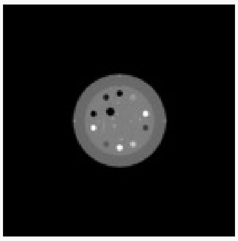

Pixel spacing x [mm]	Pixel spacing y [mm]
0.97	0.98

File name: CT.1.3.12.2.1107.5.1.4.1011.30000018020608084628100001105, series description: Thorax 3.0 B31f, module ID: CTP682,total collimation width: N/A mm, pixel size: [0.977, 0.977] mm, recon filter: B31f, scan mode: N/A

Slice thickness

Slice thickness per wire ramp [mm]

Slice thickness estimated for each of the four wires angled 23° with the in-plane

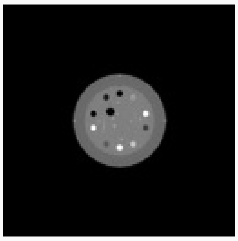

Expected slice thickness [mm]	Upper [mm]	Right [mm]	Lower [mm]	Left [mm]	Average [mm]
3.00	2.97	N/A	3.61	N/A	3.29

File name: CT.1.3.12.2.1107.5.1.4.1011.30000018020608084628100001105, series description: Thorax 3.0 B31f, module ID: CTP682, total collimation width: N/A mm , pixel size: [0.977, 0.977] mm, recon filter: B31f, scan mode: N/A

Low contrast

Contrast detail plot

Contrast detail plot, estimated by fitting to noise values for the given diameter. Each curve is derived from the fit to noise values from each column of images to the right. The fit is computed on the noise values that deviate less than 5% from the lowest values for each diameter

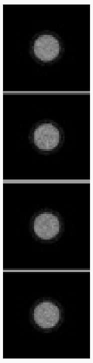

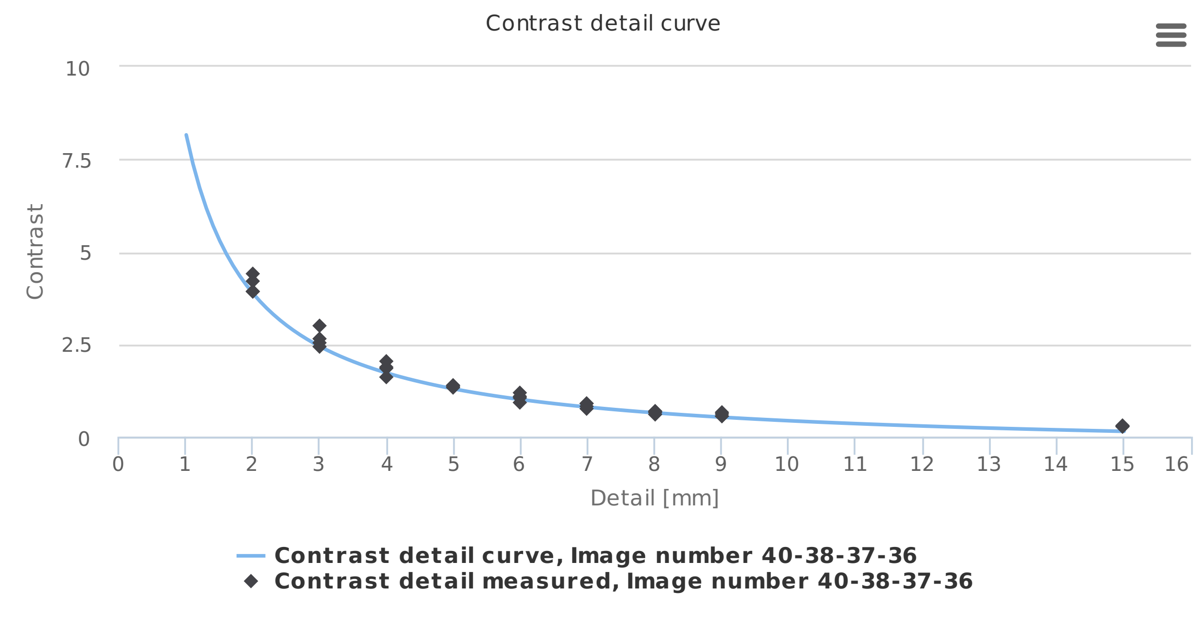

File name: CT.1.3.12.2.1107.5.1.4.1011.30000018020608084628100001130

Series description: Thorax 3.0 B31f
Module ID: CTP515
Total collimation width: N/A mm
kVp: 120
Recon FOV: 500 mm
Scan mode: N/A

Contrast detail values

Diameter (detail) of smallest detectable target for each of the three contrast values

Image number	Reconstruction filter	Tube Current*time [mAs]	Slice thickness [mm]	Detail at 1% contrast [mm]	Detail at 0.5% contrast [mm]	Detail at 0.3% contrast [mm]
40-38-37-36	B31f	20	3.0	6.0	9.0	15

Uniformity

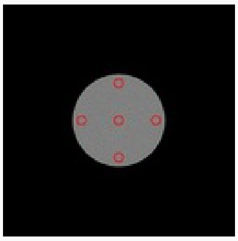

File name: **CT.1.3.12.2.1107.5.1.4.1011.30000018020608084628100001159**, series description: **Thorax 3.0 B31f**, module ID: **CTP712**, Total collimation width: **N/A mm**, kVp: **120**, mA: **37**, time: **500 ms**
recon FOV: **500 mm** recon filter: **B31f**, scan mode: **N/A**

Vertical profile and the corresponding fitted curve, 20cm module

Intensity profile across the vertical dimension of the slice and the curve fitted to the values. Start and end points are placed 1 cm from upper and lower module border, respectively.

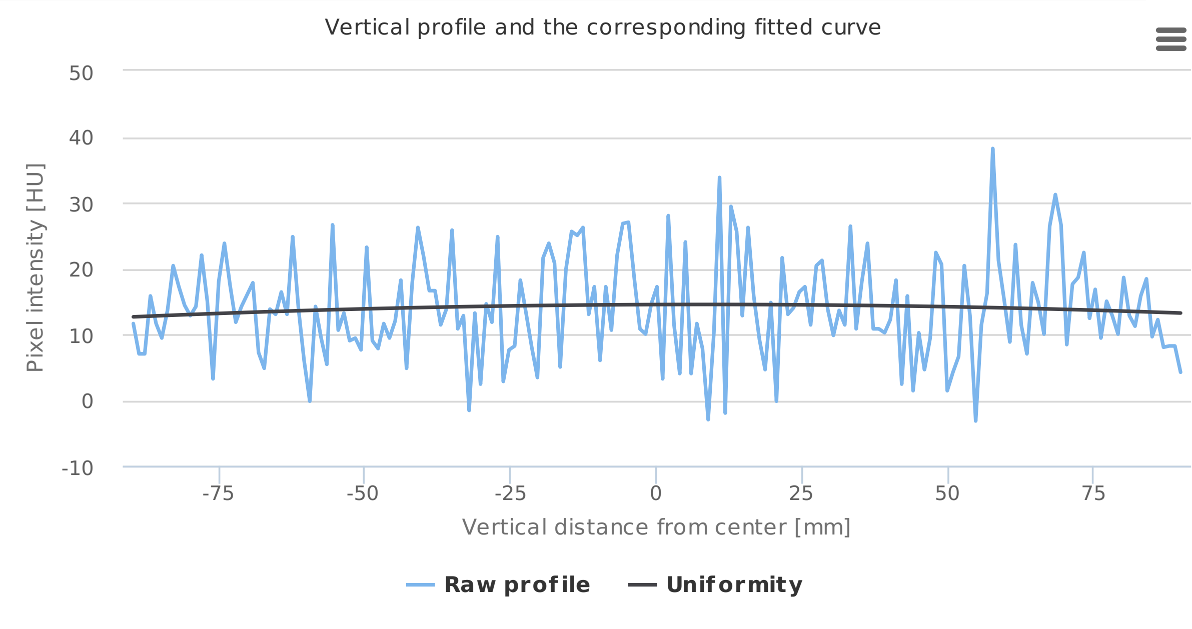

Horizontal profile and the corresponding fitted curve, 20cm module

Intensity profile across the horizontal dimension of the slice and the curve fitted to the values. Start and end points are placed 1cm from left and right module border, respectively.

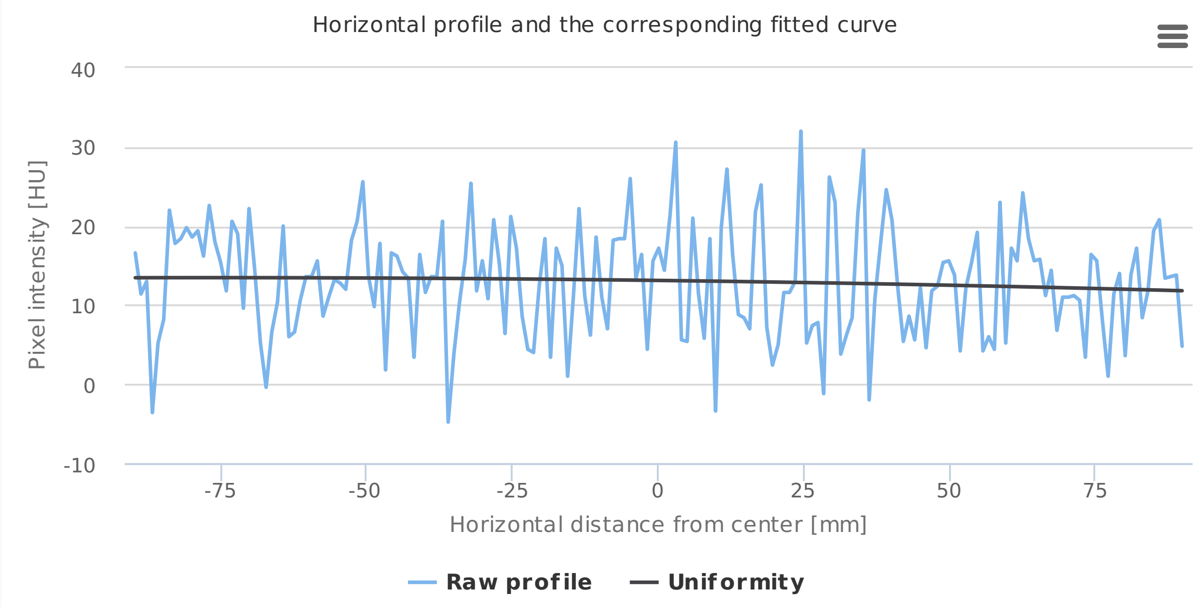

Mean CT value center region, 20cm module [HU]

Mean CT value in center region - used as reference for uniformity calculations

Image number	Reconstruction filter	Tube Current*time [mAs]	Slice thickness [mm]	Mean at center [HU]
11	B31f	18.5	3.0	14.2

Absolute differences from center in regions of interest, 20cm module [HU]

Uniformity of upper, right, lower and left regions of interest. Outer edge of each ROI is located 1cm from module border. Calculated as the absolute difference between mean CT number in the region of interest and the mean CT number in the center region.

Image number	Reconstruction filter	Tube Current*time [mAs]	Slice thickness [mm]	Upper ROI [HU]	Right ROI [HU]	Lower ROI [HU]	Left ROI [HU]
11	B31f	18.5	3.0	0.7	1.1	1.6	1.9

Noise, 20cm module [HU]

Noise in the center region (with diameter 40% of the diameter of the module) computed as the standard deviation of the CT numbers

Image number	Reconstruction filter	Tube Current*time [mAs]	Slice thickness [mm]	Noise [HU]
11	B31f	18.5	3.0	20.1

Noise and mean values plot, 20cm module

Noise and mean CT values displayed in a bar plot

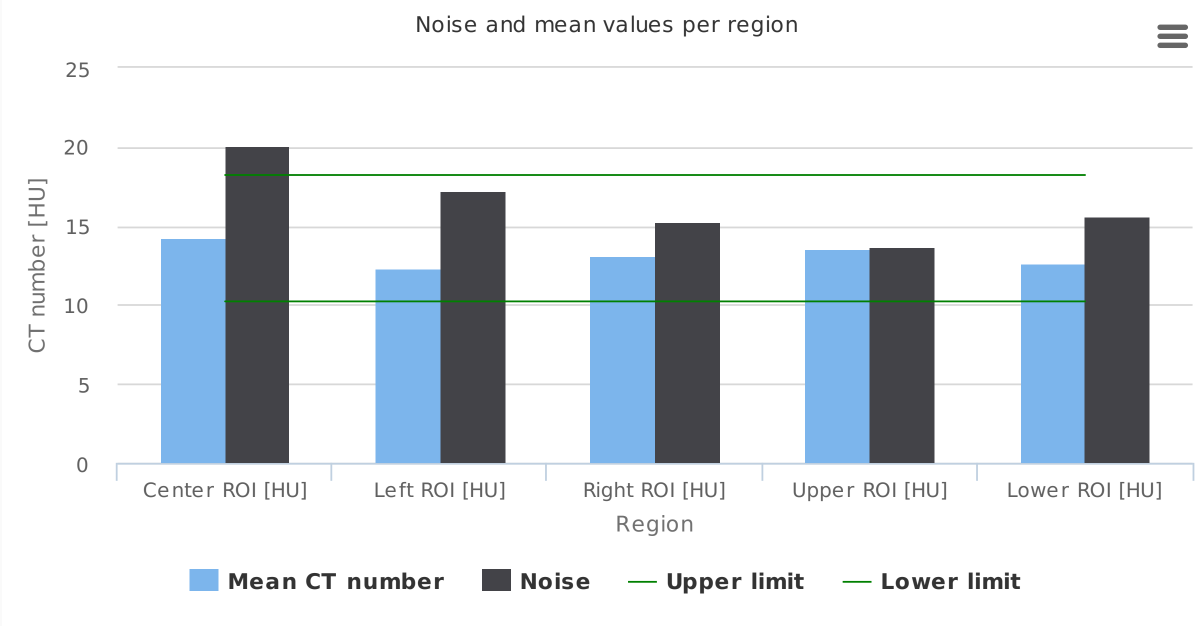

Positional

Center of phantom [pixels]

(x,y) Center of Phantom

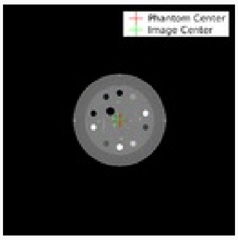

Center x-coordinate	Center y-coordinate
258	257

Rotation [°]

Rotation of phantom around z-axis

-0.045

Tilt [°]

Tilt, rotation around x-axis

1.1

Yaw [°]

Yaw, rotation around y-axis

1.4

Warnings

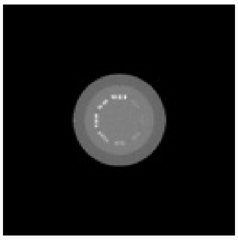

Slices from the CTP714 module were identified. Currently, the service is not reporting information on the high resolution gauges as it is a subjective visual test. Please upload a slice from the CTP682 module including the MTF bead.

File name: CT.1.3.12.2.1107.5.1.4.1011.30000018020608084628100001118
Series description: Thorax 3.0 B31f
Module ID: CTP714
Total collimation width: N/A mm
mA: 39
Time: 500
kVp: 120
Slice thickness: 3.00 mm
Scan mode: N/A

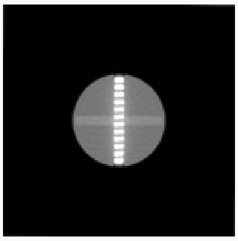

The wave harmonics analysis requires slice thickness to be less than 2mm. Please upload a thinner slice.

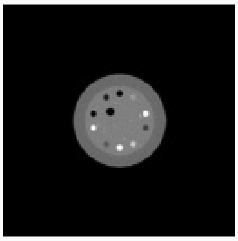

File name: CT.1.3.12.2.1107.5.1.4.1011.30000018020608084628100001104
Series description: Thorax 3.0 B31f
Module ID: CTP682
Total collimation width: N/A mm
mA: 39
Time: 500
kVp: 120
Slice thickness: 3.00 mm
Scan mode: N/A

Slices from the CTP682 module were detected. PSF is undersampled and hence no results are reported for the module. Please consider using a smaller reconstructed FOV. Scanning protocol recommendations are available in our help pages.

---

imageOwl Catphan® QA

Petros Kalendralis |

View report

Radboudumc *2018-06-06 09:13:18*

Philips Brilliance Big Bore / Petros Kalendralis

Sensitometry

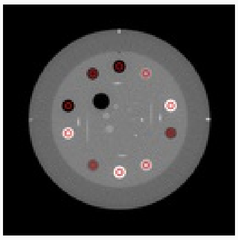

File name: **1.2.840.113704.1.111.2252.1521043348.8_0002_000010_1521045540131a**, series description: **THORAX**, module ID: **CTP682**, Total collimation width: **N/A mm**, kVp: **120**, mA: **134**, time: **923 ms** slice thickness: **3.00 mm**, recon FOV: **255 mm**, recon filter: **B**, scan mode: **HELIX**

Linearity plot

CT number vs attenuation coefficient

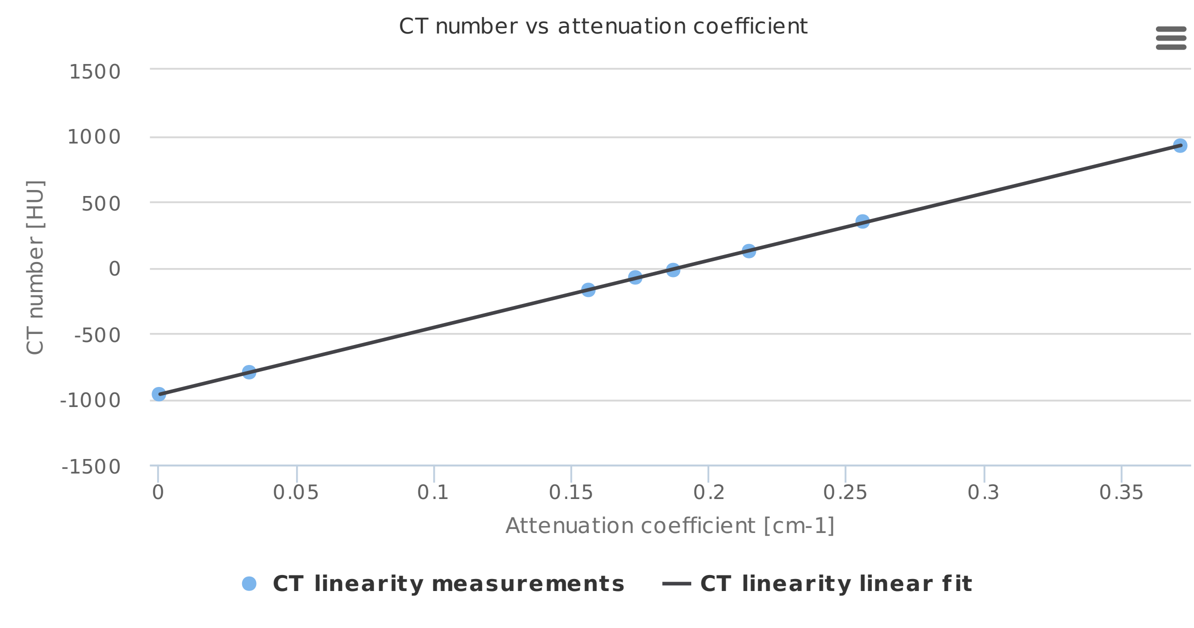

Air [HU]

Measured CT number of air

-965

Lung foam [HU]

Measured CT number of lung foam

-798

PMP [HU]
Measured CT number of PMP

-173

LDPE [HU]
Measured CT number of LDPE

-78

Polystyrene [HU]
Measured CT number of polystyrene

-22

Acrylic [HU]
Measured CT number of acrylic

122

Hydroxyapatite 20% [HU]
Measured CT number of 20% hydroxyapatite

237

Delrin [HU]
Measured CT number of delrin

347

Hydroxyapatite 50% [HU]
Measured CT number of 50% hydroxyapatite

663

Teflon [HU]
Measured CT number of teflon

923

Contrast scale [$cm^{-1}HU^{-1}$]
Contrast scale, slope of linearity plot

0.000196

Effective energy
Effective energy calculated by fitting to table values for keV and attenuation coeffcients

71

Residual plot
Total residuals for fitting table keV values to measured values

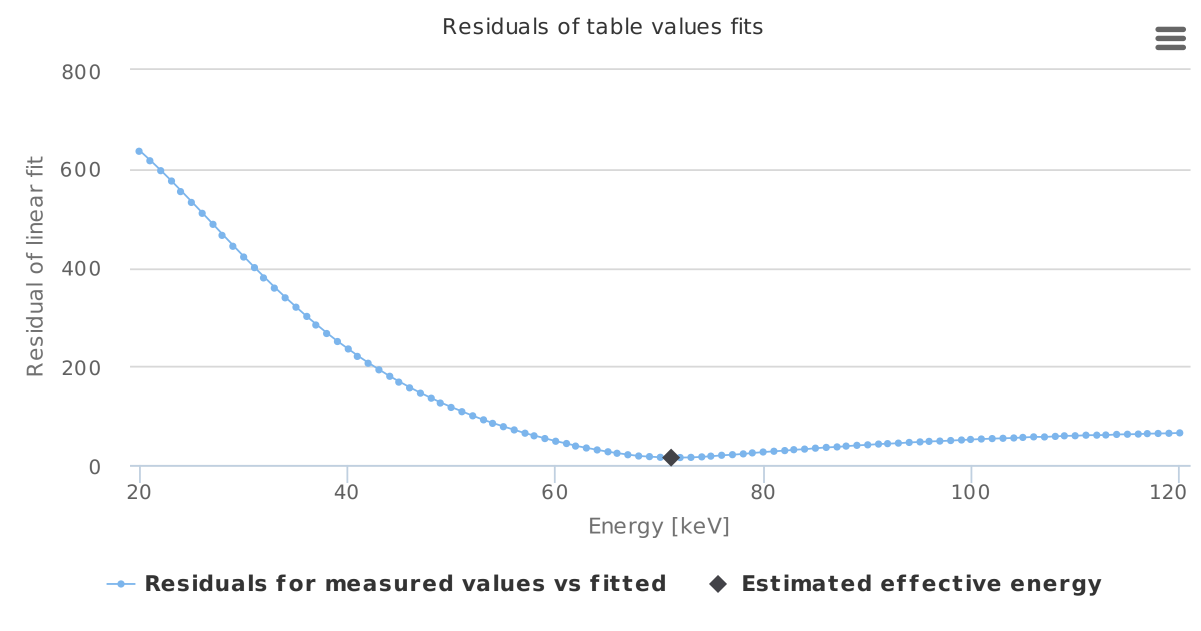

Sensitometry plot

CT numbers vs estimated keV from table for each material

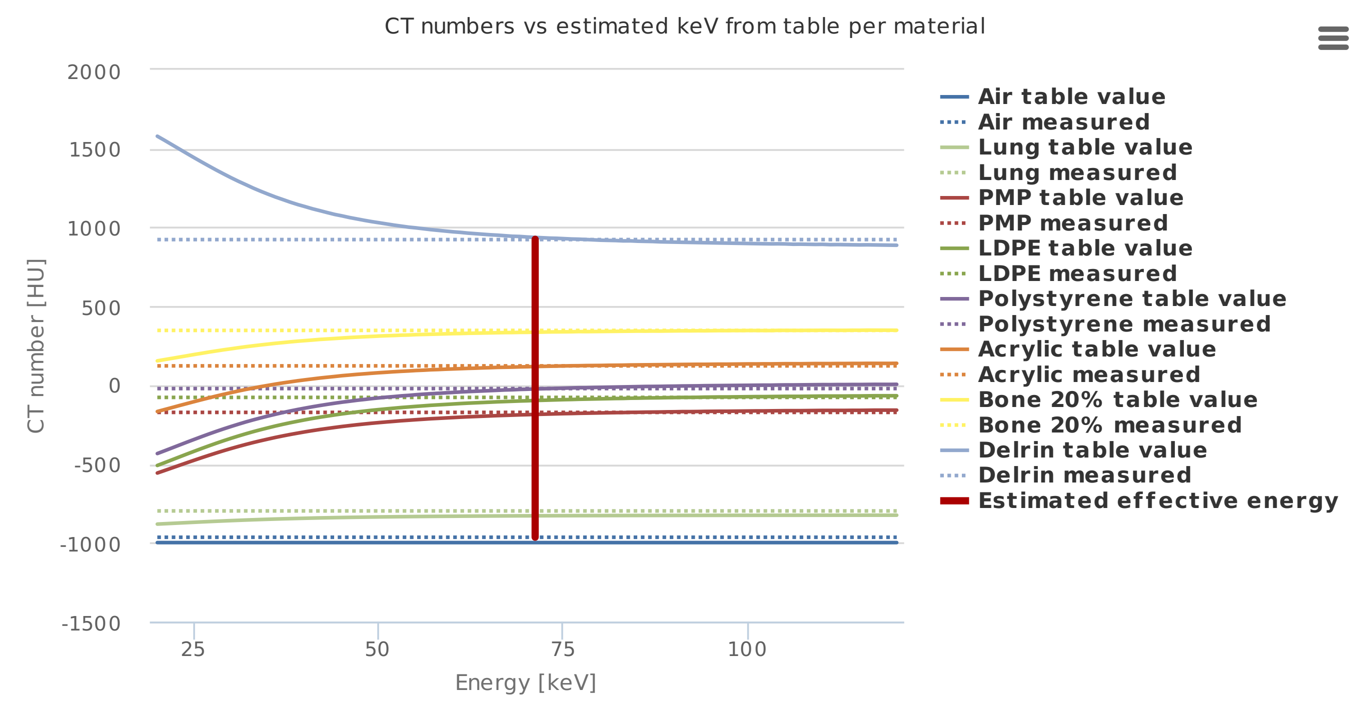

Spatial linearity

Measured pixel spacing [mm]

Measured pixel spacing

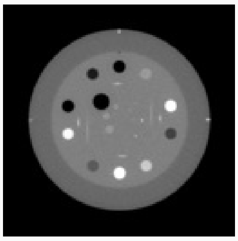

Pixel spacing x [mm]	Pixel spacing y [mm]
0.25	0.25

File name: 1.2.840.113704.1.111.2252.1521043348.8_0002_000010_1521045540131a, series description: THORAX, module ID: CTP682,total collimation width: N/A mm, pixel size: [0.249, 0.249] mm, recon filter: B, scan mode: HELIX

Slice thickness

Slice thickness per wire ramp [mm]

Slice thickness estimated for each of the four wires angled 23° with the in-plane

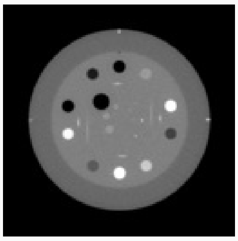

Expected slice thickness [mm]	Upper [mm]	Right [mm]	Lower [mm]	Left [mm]	Average [mm]
3.00	3.04	N/A	3.03	N/A	3.04

File name: 1.2.840.113704.1.111.2252.1521043348.8_0002_000010_1521045540131a, series description: THORAX, module ID: CTP682, total collimation width: N/A mm , pixel size: [0.249, 0.249] mm, recon filter: B, scan mode: HELIX

MTF

Modulation transfer function (MTF) from upper bead

Plot of modulation transfer function for upper bead

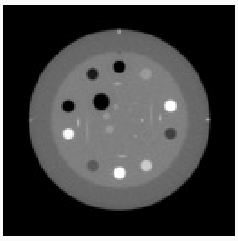

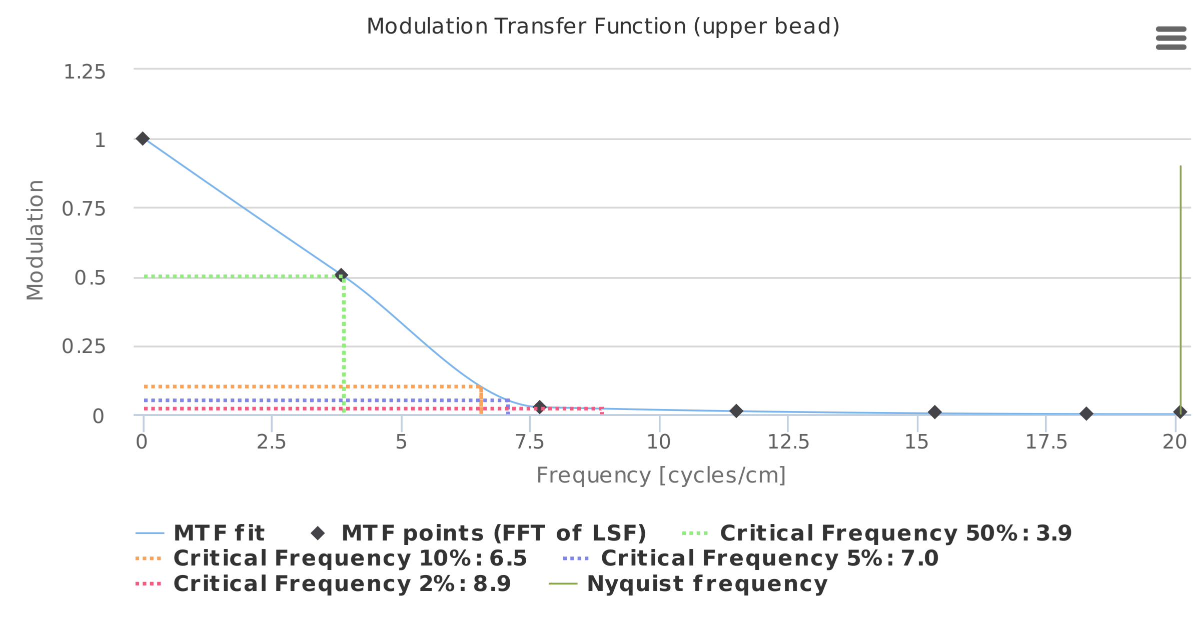

File name: 1.2.840.113704.1.111.2252.1521043348.8_0002_000010_1521045540131a
Series description: THORAX
Module ID: CTP682
Total collimation width: N/A mm
mA: 134
Time: 923
kVp: 120
Slice thickness: 3.00 mm

Scan mode: HELIX

Line spread function (LSF) from upper bead

Plot of line spread function for upper bead

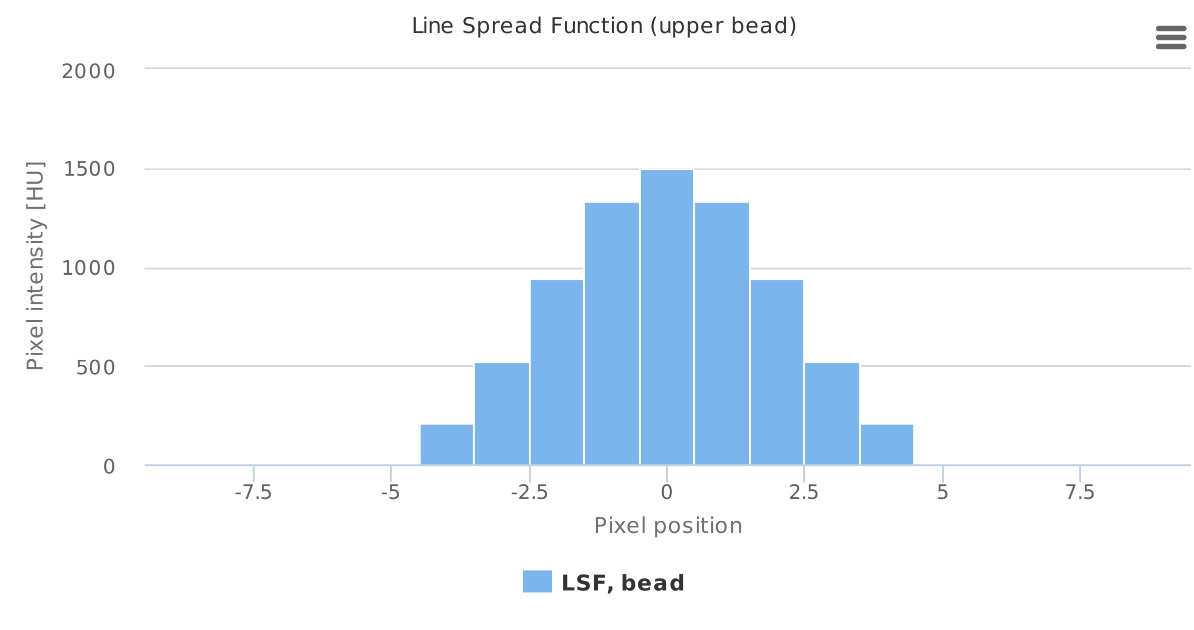

Critical frequency upper bead [cycles/cm]

Critical frequency values for upper bead for 50%,10%, 5%, and 2% of MTF

Reconstruction filter	Field of View [mm]	50%	10%	5%	2%
B	255	3.87	6.53	7.05	8.87

Modulation transfer function (MTF) from wire

Plot of modulation transfer function for the 50 µm teflon wire

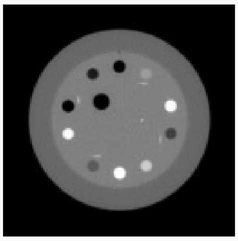

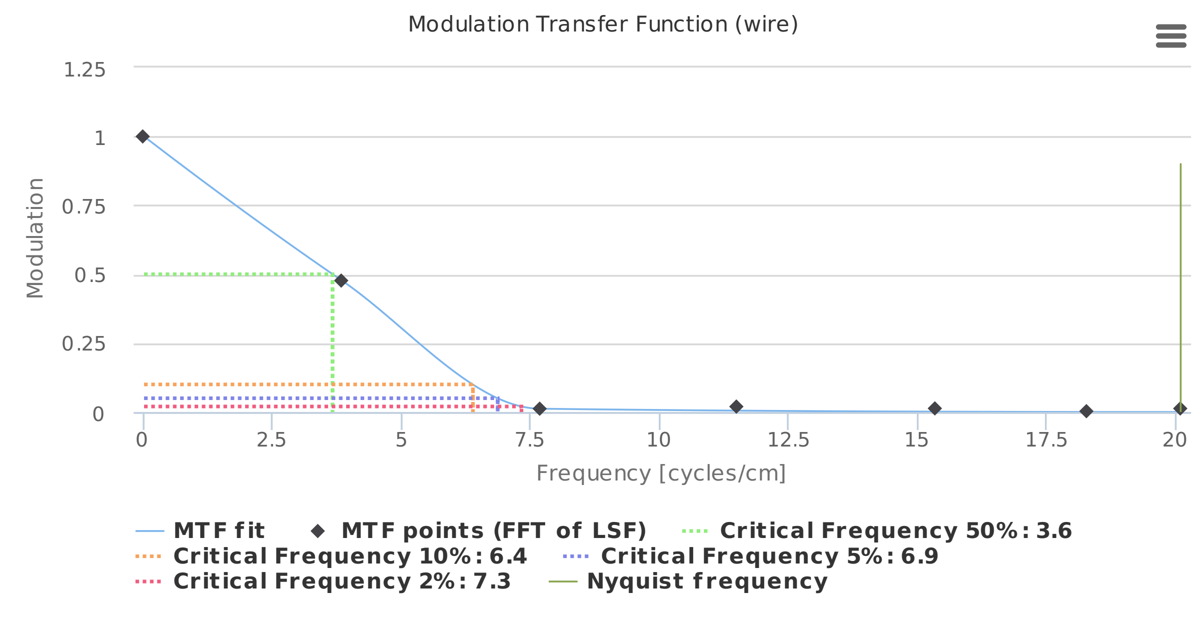

File name: 1.2.840.113704.1.111.2252.1521043348.8_0002_000014_1521045541131e
Series description: THORAX
Module ID: CTP682
Total collimation width: N/A mm
mA: 134
Time: 923
kVp: 120
Slice thickness: 3.00 mm
Scan mode: HELIX

Line spread function (LSF) from wire

Plot of line spread function for the 50 µm teflon wire

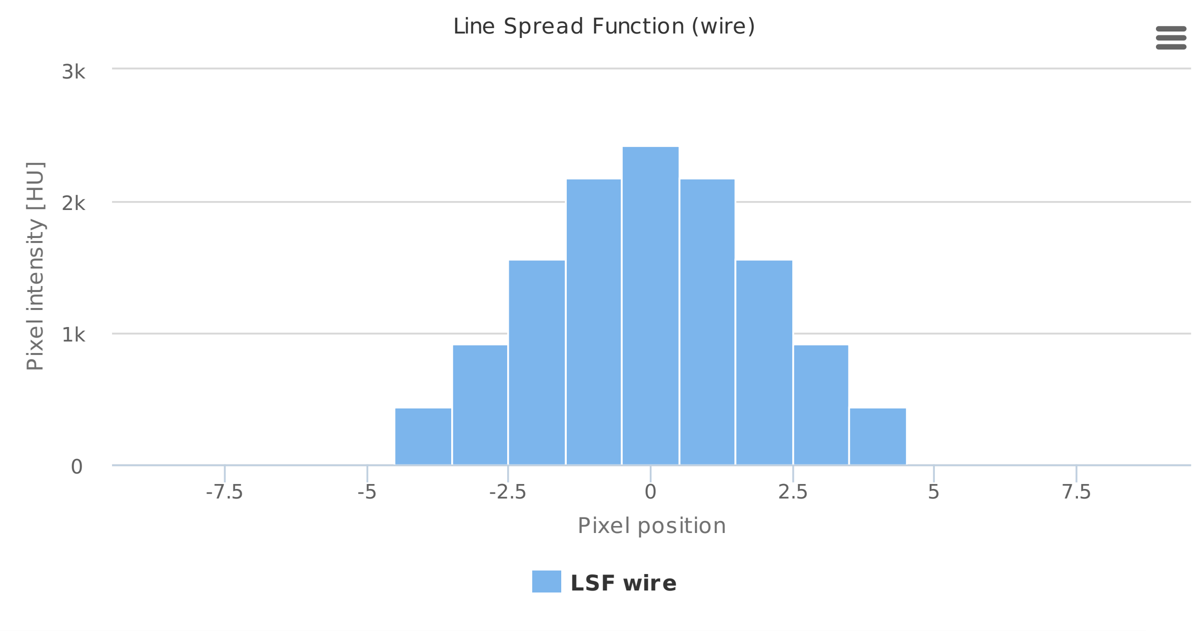

Critical frequency wire [cycles/cm]

Critical frequency values for wire for 50%,10%, 5%, and 2% of MTF

Reconstruction filter	Field of View [mm]	50%	10%	5%	2%
B	255	3.65	6.37	6.85	7.31

Uniformity

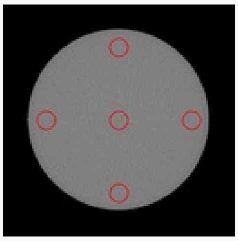

File name: **1.2.840.113704.1.111.2252.1521043348.8_0002_000064_15210455511350**, series description: **THORAX**, module ID: **CTP712**, Total collimation width: **N/A mm**, kVp: **120**, mA: **134**, time: **923 ms**
recon FOV: **255 mm** recon filter: **B**, scan mode: **HELIX**

Vertical profile and the corresponding fitted curve, 20cm module

Intensity profile across the vertical dimension of the slice and the curve fitted to the values. Start and end points are placed 1 cm from upper and lower module border, respectively.

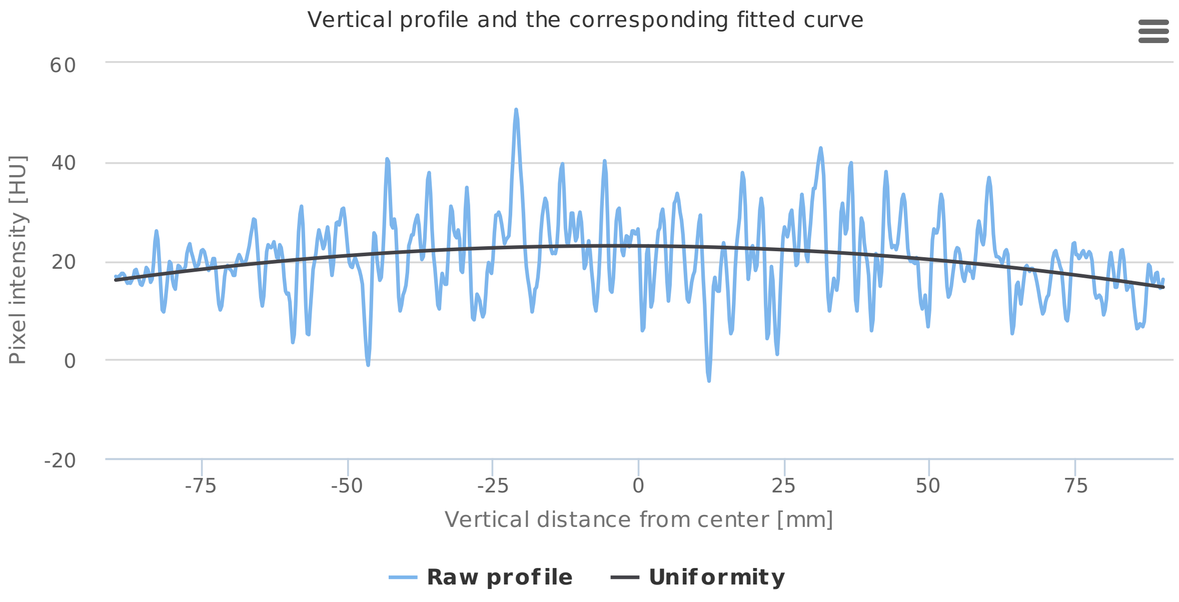

Horizontal profile and the corresponding fitted curve, 20cm module

Intensity profile across the horizontal dimension of the slice and the curve fitted to the values. Start and end points are placed 1cm from left and right module border, respectively.

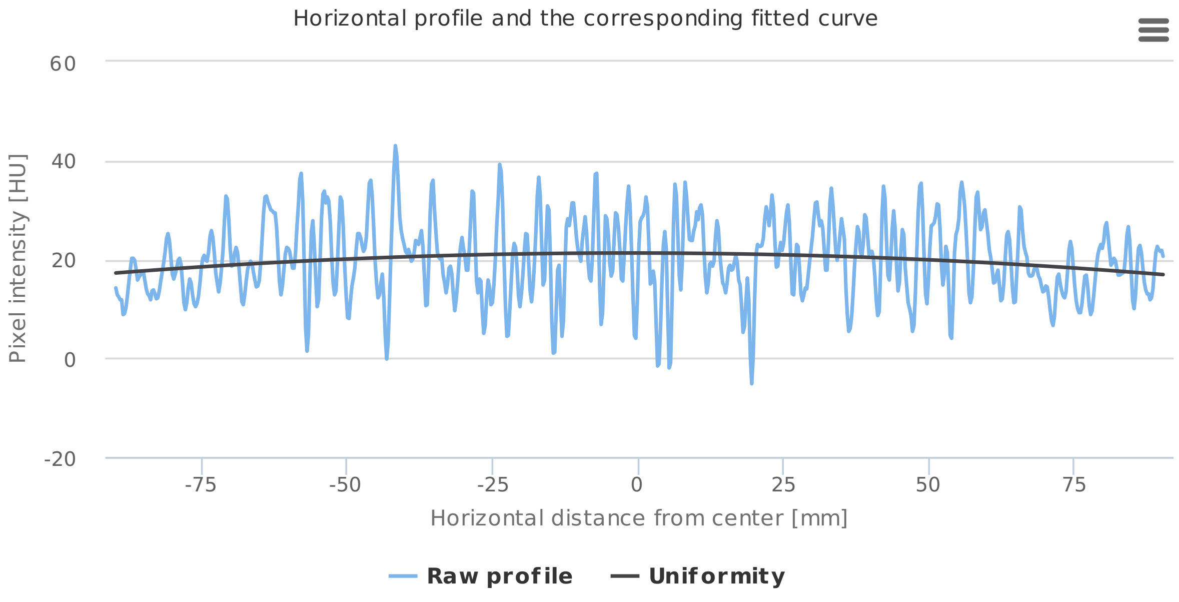

Mean CT value center region, 20cm module [HU]

Mean CT value in center region - used as reference for uniformity calculations

Image number	Reconstruction filter	Tube Current*time [mAs]	Slice thickness [mm]	Mean at center [HU]
64	B	123.7	3.0	20.1

Absolute differences from center in regions of interest, 20cm module [HU]

Uniformity of upper, right, lower and left regions of interest. Outer edge of each ROI is located 1cm from module border. Calculated as the absolute difference between mean CT number in the region of interest and the mean CT number in the center region.

Image number	Reconstruction filter	Tube Current*time [mAs]	Slice thickness [mm]	Upper ROI [HU]	Right ROI [HU]	Lower ROI [HU]	Left ROI [HU]
64	B	123.7	3.0	2.2	3.0	2.5	2.4

Noise, 20cm module [HU]

Noise in the center region (with diameter 40% of the diameter of the module) computed as the standard deviation of the CT numbers

Image number	Reconstruction filter	Tube Current*time [mAs]	Slice thickness [mm]	Noise [HU]
64	B	123.7	3.0	10.5

Noise and mean values plot, 20cm module

Noise and mean CT values displayed in a bar plot

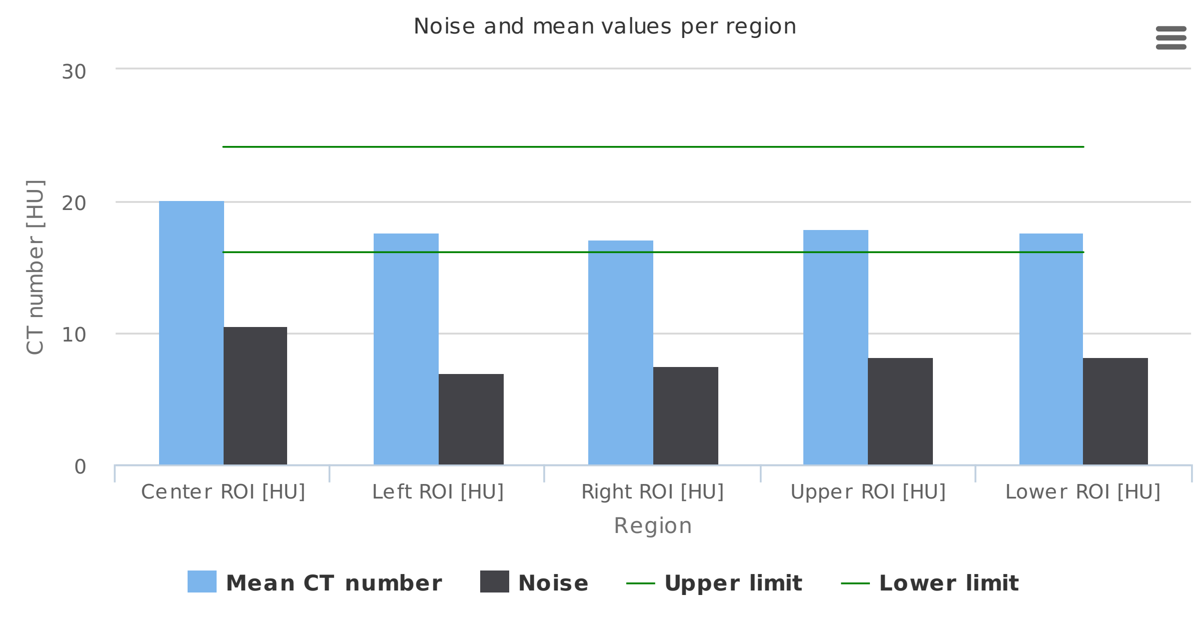

Wave

In-plane MTF from bead

In-plane MTF plots from all beads

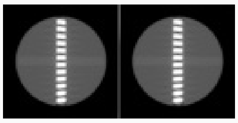

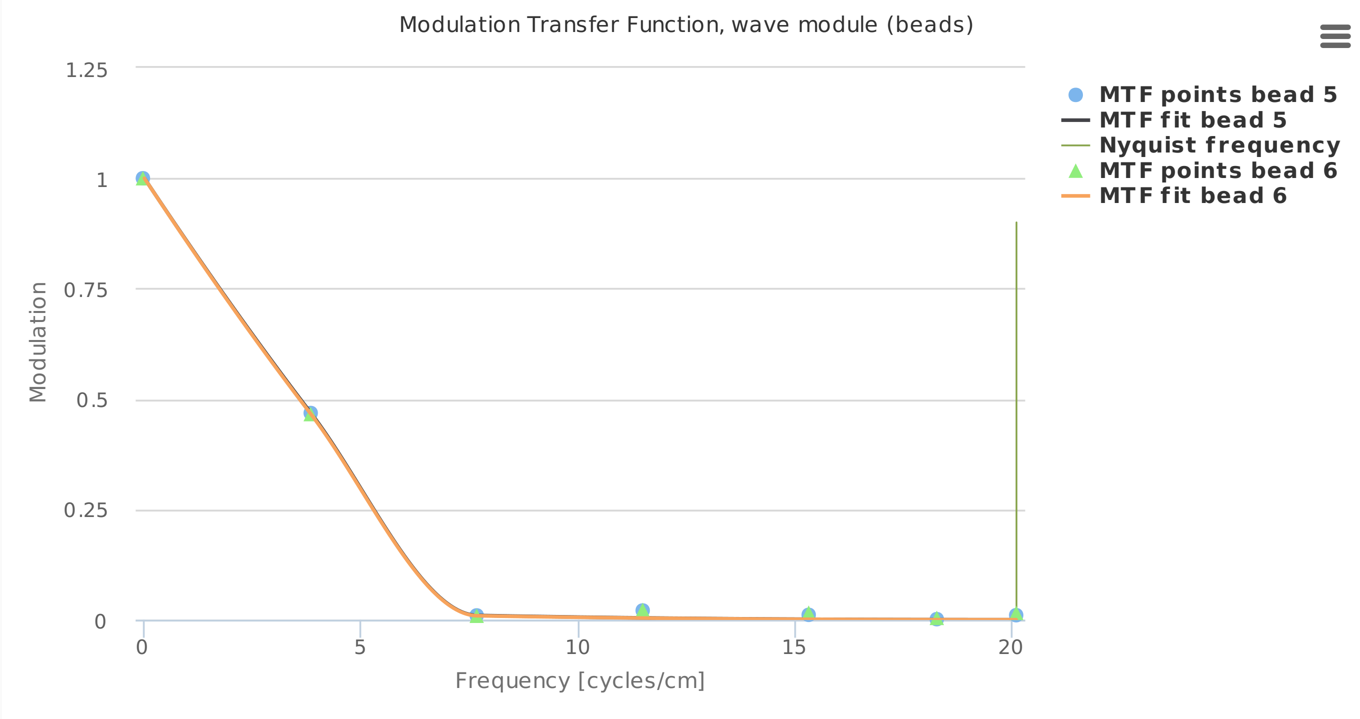

File name: 1.2.840.113704.1.111.2252.1521043348.8_0002_000050_15210455481342
Series description: THORAX
module ID: CTP721
total collimation width: N/A mm
mA: 134
time: 923
slice thickness: 3.00
recon FOV: 255 mm
recon filter: B
scan mode: HELIX

Critical frequencies, beads, wave module [cycles/cm]

Critical frequency values for all detected beads for 50%,10%, 5%, and 2% of MTF

Bead number	Reconstruction filter	Field of View [mm]	50%	10%	5%	2%
5	B	255	3.6	6.3	6.8	7.2
6	B	255	3.6	6.3	6.8	7.2

Positional

Center of phantom [pixels]

(x,y) Center of Phantom

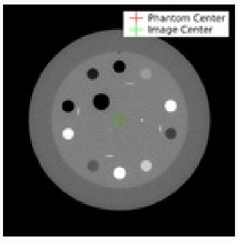

Center x-coordinate	Center y-coordinate
514	510

Rotation [°]

Rotation of phantom around z-axis

-0.3

Tilt [°]
Tilt, rotation around x-axis

-0.14

Yaw [°]
Yaw, rotation around y-axis

-0.11

Warnings

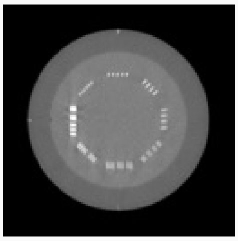

Slices from the CTP714 module were identified. Currently, the service is not reporting information on the high resolution gauges as it is a subjective visual test. Please upload a slice from the CTP682 module including the MTF bead.

File name: 1.2.840.113704.1.111.2252.1521043348.8_0002_000023_15210455431327
Series description: THORAX
Module ID: CTP714
Total collimation width: N/A mm
mA: 134
Time: 923
kVp: 120
Slice thickness: 3.00 mm
Scan mode: HELIX

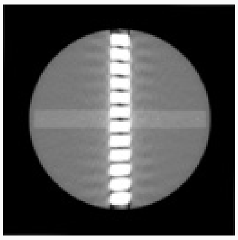

The wave harmonics analysis requires slice thickness to be less than 2mm. Please upload a thinner slice.

support@imageowl.com / Terms of use / Copyright © 2018 Image Owl

Petros Kalendralis |

View report

UMCG/ PC2 2018-08-20 10:22:53

SIEMENS Biograph 64 1005 / Petros Kalendralis

Sensitometry

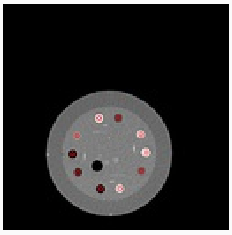

File name: **CATPHAN.CT.THORAX_THORAX_1_(ADULT).0001.0066.2018.07.11.18.06.26.501374.150368070**, series description: **Thorax + C 3.0 I30f 2**, module ID: **CTP682**, Total collimation width: **1.920000e+01 mm**, kVp: **80**, mA: **221**, time: **500 ms** slice thickness: **3.00 mm**, recon FOV: **360 mm**, recon filter: **I30f2**, scan mode: **N/A**

Linearity plot

CT number vs attenuation coefficient

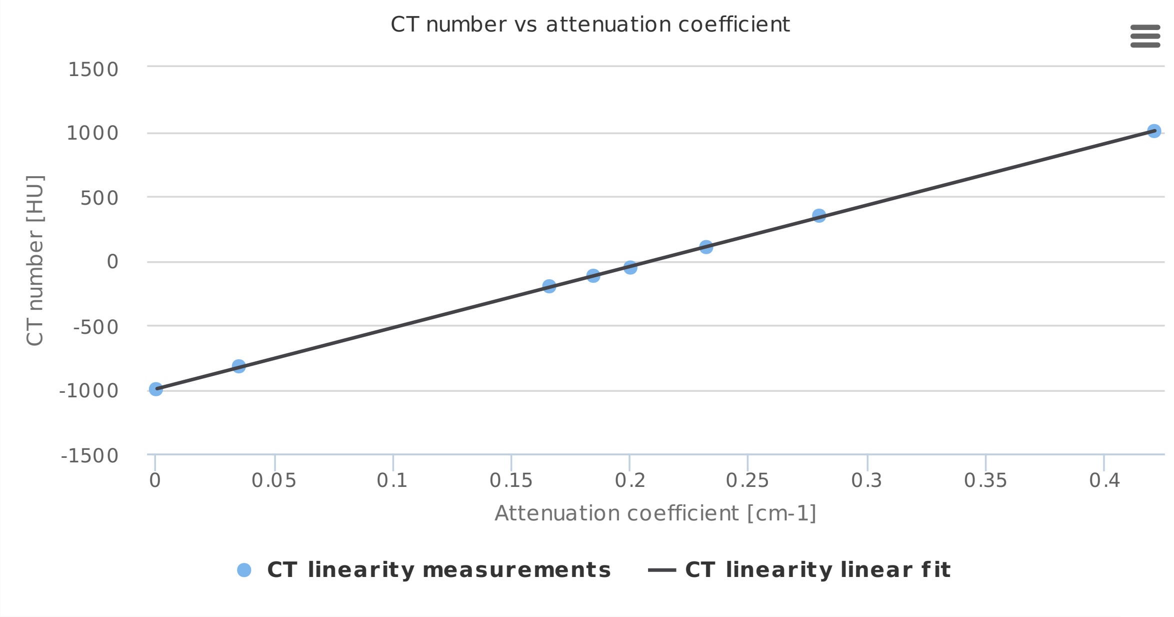

Air [HU]

Measured CT number of air

-1000

Lung foam [HU]

Measured CT number of lung foam

-822

PMP [HU]
Measured CT number of PMP

-202

LDPE [HU]
Measured CT number of LDPE

-120

Polystyrene [HU]
Measured CT number of polystyrene

-56

Acrylic [HU]
Measured CT number of acrylic

103

Hydroxyapatite 20% [HU]
Measured CT number of 20% hydroxyapatite

307

Delrin [HU]
Measured CT number of delrin

346

Hydroxyapatite 50% [HU]
Measured CT number of 50% hydroxyapatite

918

Teflon [HU]
Measured CT number of teflon

1004

Contrast scale [$cm^{-1}HU^{-1}$]
Contrast scale, slope of linearity plot

0.00021

Effective energy
Effective energy calculated by fitting to table values for keV and attenuation coeffcients

57

Residual plot
Total residuals for fitting table keV values to measured values

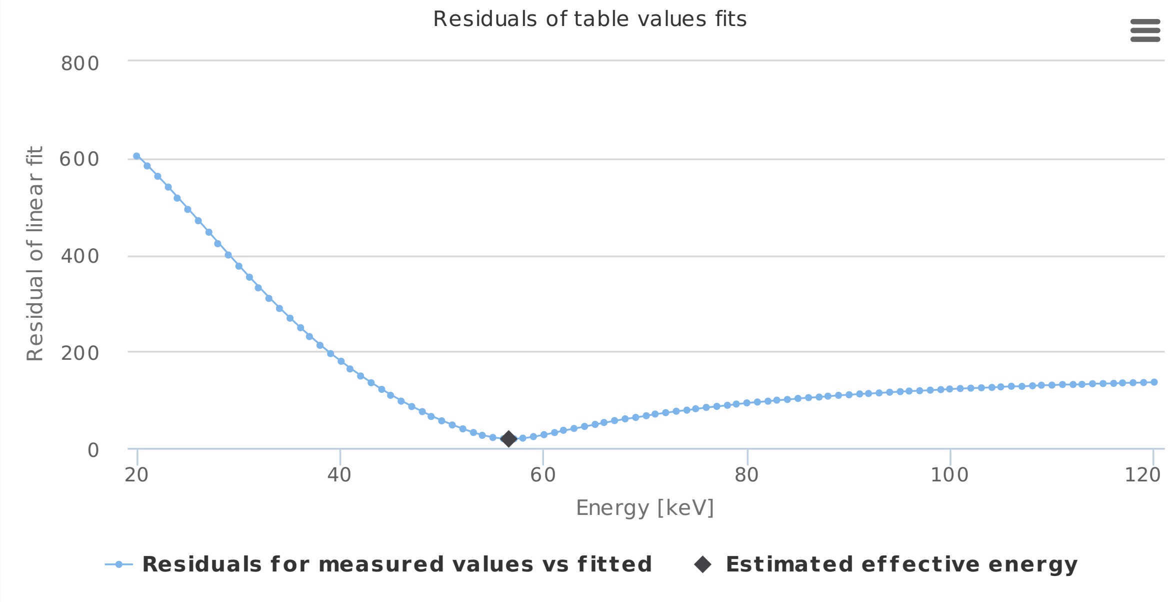

Sensitometry plot

CT numbers vs estimated keV from table for each material

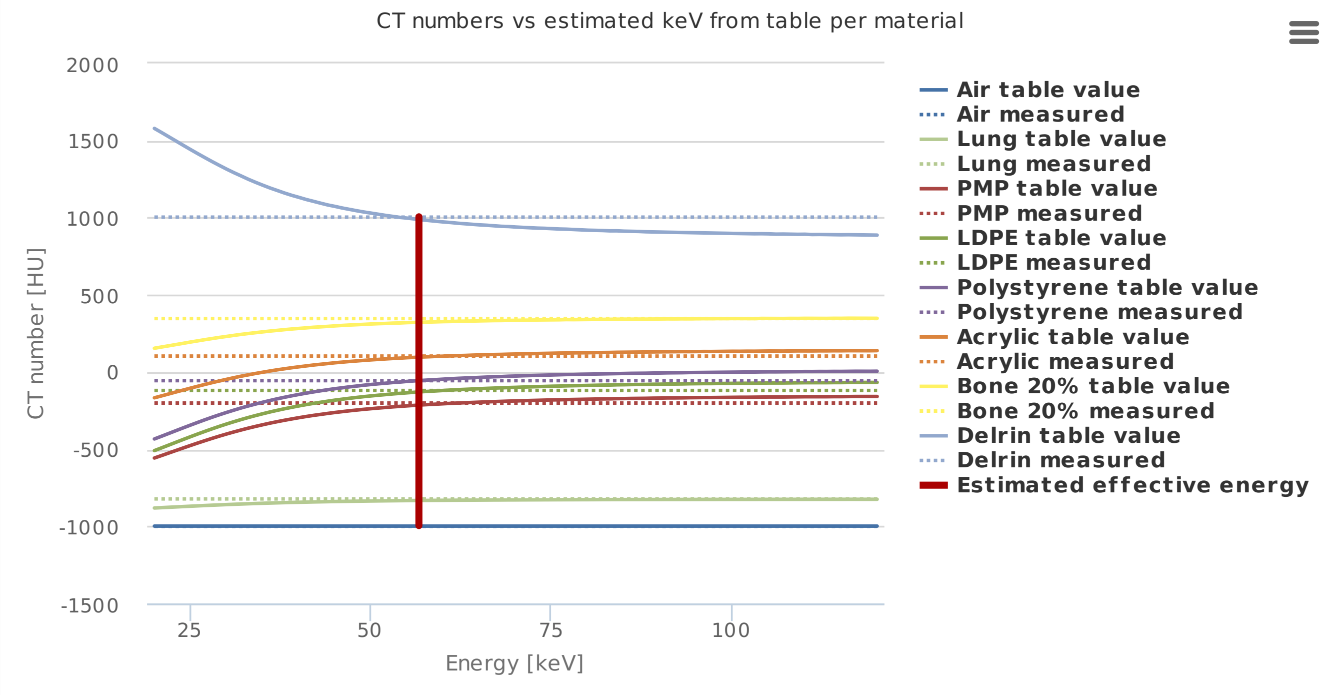

Spatial linearity

Measured pixel spacing [mm]

Measured pixel spacing

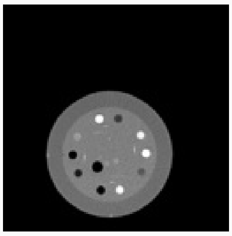

Pixel spacing x [mm]	Pixel spacing y [mm]
28.92	37.34

File name: CATPHAN.CT.THORAX_THORAX_1_(ADULT).0001.0066.2018.07.11.18.06.26.501374.150368070, series description: Thorax + C 3.0 I30f 2, module ID: CTP682,total collimation width: 1.920000e+01 mm, pixel size: [0.703, 0.703] mm, recon filter: I30f2, scan mode: N/A

Slice thickness

Slice thickness per wire ramp [mm]

Slice thickness estimated for each of the four wires angled 23° with the in-plane

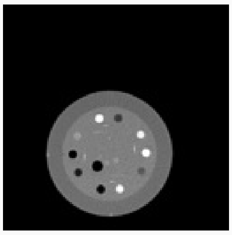

Expected slice thickness [mm]	Upper [mm]	Right [mm]	Lower [mm]	Left [mm]	Average [mm]
3.00	2.62	N/A	2.82	N/A	2.72

File name: CATPHAN.CT.THORAX_THORAX_1_(ADULT).0001.0066.2018.07.11.18.06.26.501374.150368070, series description: Thorax + C 3.0 I30f 2, module ID: CTP682, total collimation width: 1.920000e+01 mm , pixel size: [0.703, 0.703] mm, recon filter: I30f2, scan mode: N/A

Low contrast

Contrast detail plot

Contrast detail plot, estimated by fitting to noise values for the given diameter. Each curve is derived from the fit to noise values from each column of images to the right. The fit is computed on the noise values that deviate less than 5% from the lowest values for each diameter

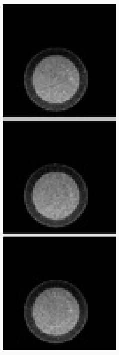

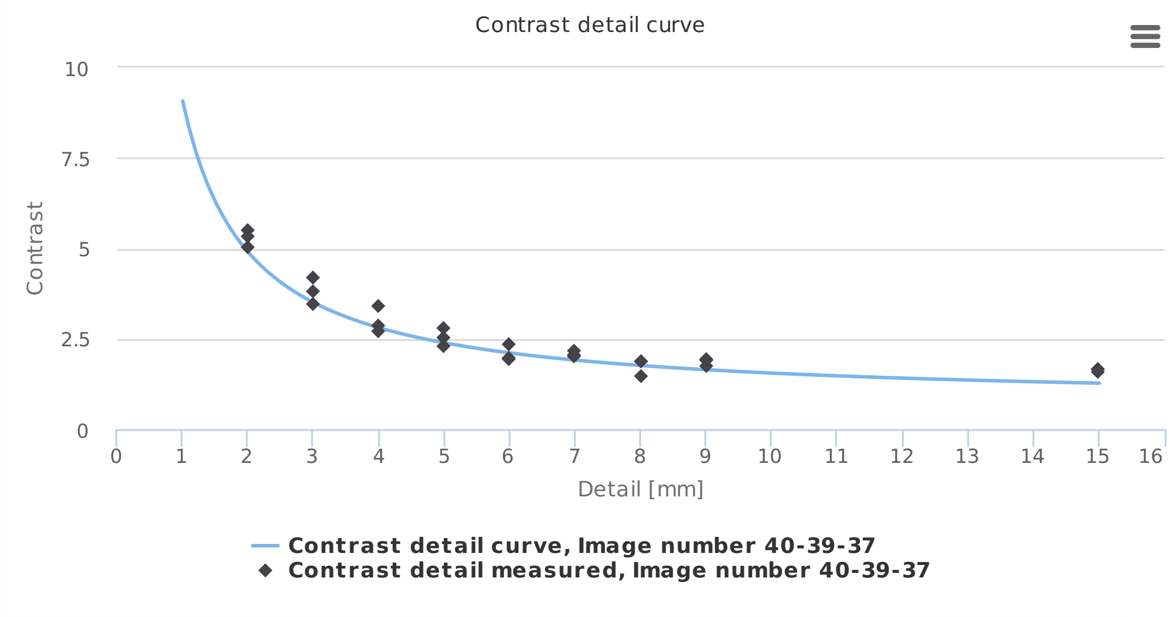

File name: CATPHAN.CT.THORAX_THORAX_1_(ADULT).0001.0040.2018.07.11.18.06.26.501374.150367368
Series description: Thorax + C 3.0 I30f 2
Module ID: CTP515

Total collimation width: 1.920000e+01 mm
kVp: 80
Recon FOV: 360 mm
Scan mode: N/A

Contrast detail values

Diameter (detail) of smallest detectable target for each of the three contrast values

Image number	Reconstruction filter	Tube Current*time [mAs]	Slice thickness [mm]	Detail at 1% contrast [mm]	Detail at 0.5% contrast [mm]	Detail at 0.3% contrast [mm]
40-39-37	I30f2	73	3.0	>15	>15	>15

Uniformity

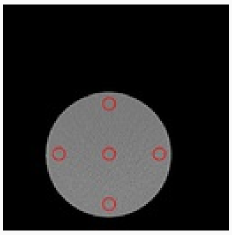

File name: **CATPHAN.CT.THORAX_THORAX_1_(ADULT).0001.0012.2018.07.11.18.06.26.501374.150366612**, series description: **Thorax + C 3.0 I30f 2**, module ID: **CTP712**, Total collimation width: **1.920000e+01 mm**, kVp: **80**, mA: **163**, time: **500 ms** recon FOV: **360 mm** recon filter: **I30f2**, scan mode: **N/A**

Vertical profile and the corresponding fitted curve, 20cm module

Intensity profile across the vertical dimension of the slice and the curve fitted to the values. Start and end points are placed 1 cm from upper and lower module border, respectively.

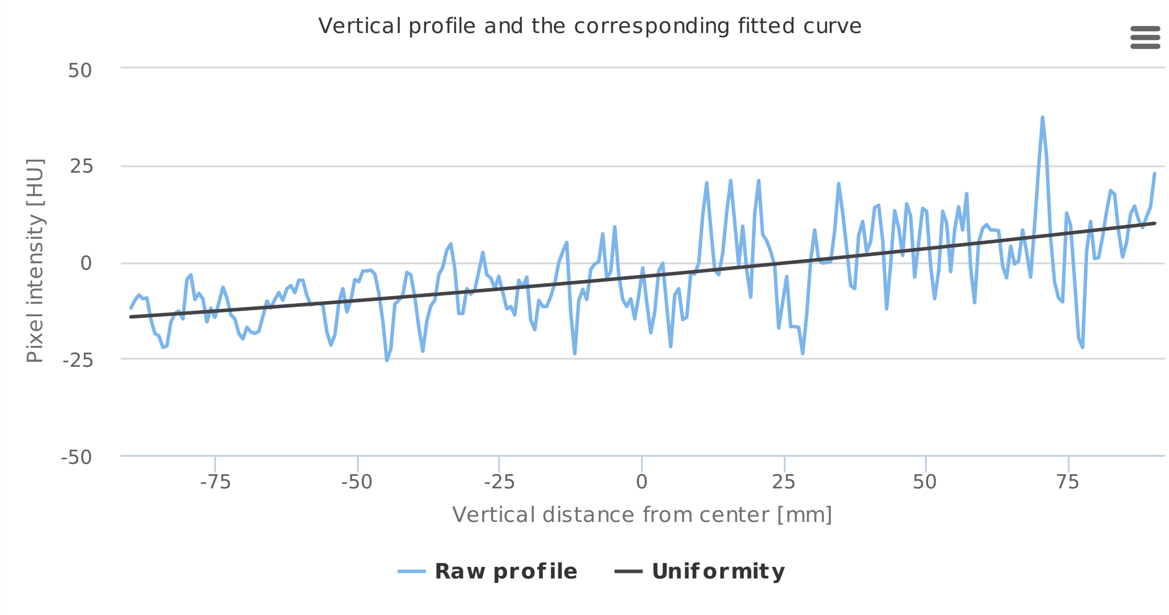

Horizontal profile and the corresponding fitted curve, 20cm module

Intensity profile across the horizontal dimension of the slice and the curve fitted to the values. Start and end points are placed 1cm from left and right module border, respectively.

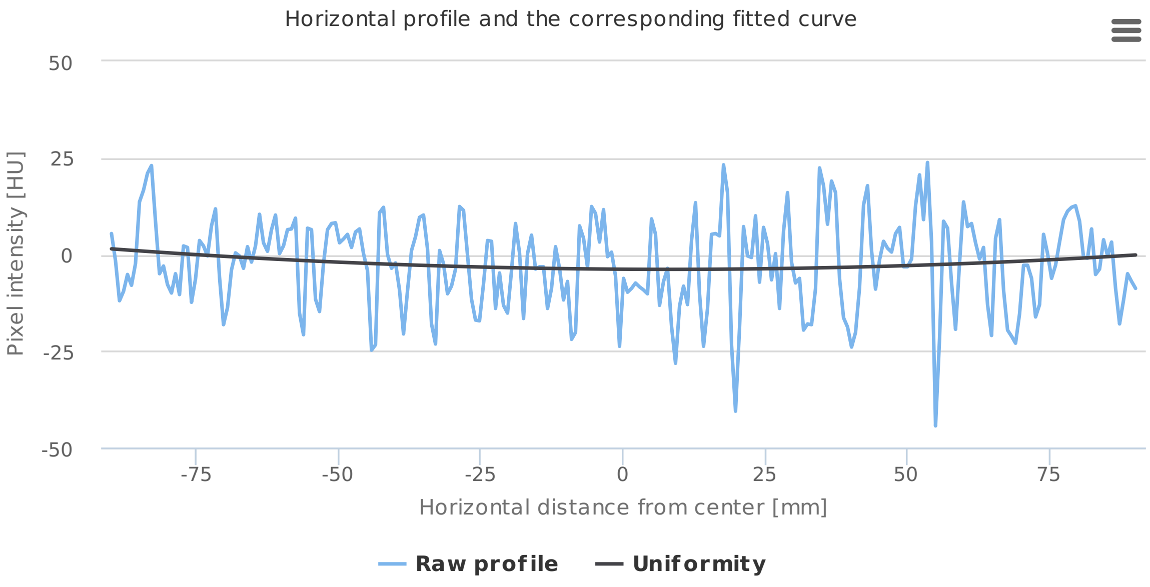

Mean CT value center region, 20cm module [HU]

Mean CT value in center region - used as reference for uniformity calculations

Image number	Reconstruction filter	Tube Current*time [mAs]	Slice thickness [mm]	Mean at center [HU]
12	I30f2	81.5	3.0	-5.7

Absolute differences from center in regions of interest, 20cm module [HU]

Uniformity of upper, right, lower and left regions of interest. Outer edge of each ROI is located 1cm from module border. Calculated as the absolute difference between mean CT number in the region of interest and the mean CT number in the center region.

Image number	Reconstruction filter	Tube Current*time [mAs]	Slice thickness [mm]	Upper ROI [HU]	Right ROI [HU]	Lower ROI [HU]	Left ROI [HU]
12	I30f2	81.5	3.0	7.1	5.1	13.2	3.9

Noise, 20cm module [HU]

Noise in the center region (with diameter 40% of the diameter of the module) computed as the standard deviation of the CT numbers

Image number	Reconstruction filter	Tube Current*time [mAs]	Slice thickness [mm]	Noise [HU]
12	I30f2	81.5	3.0	18.0

Noise and mean values plot, 20cm module

Noise and mean CT values displayed in a bar plot

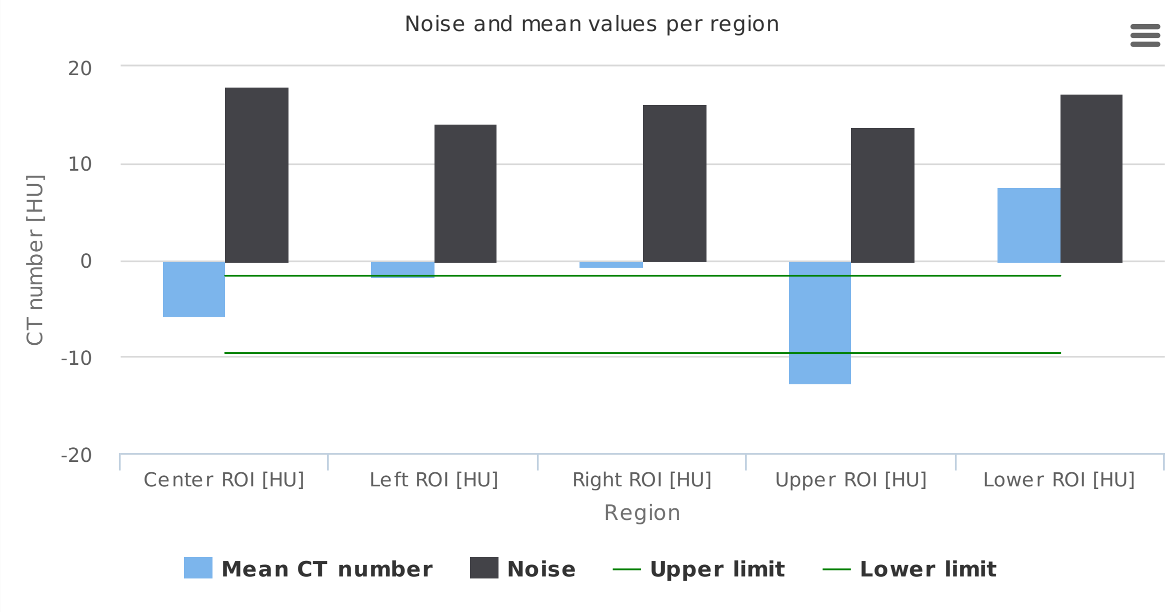

Positional

Center of phantom [pixels]

(x,y) Center of Phantom

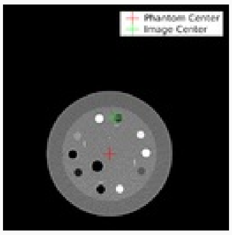

Center x-coordinate	Center y-coordinate
242	338

Rotation [°]

Rotation of phantom around z-axis

2.9

Tilt [°]

Tilt, rotation around x-axis

-0.87

Yaw [°]

Yaw, rotation around y-axis

-0.94

Metal Artefact Analysis

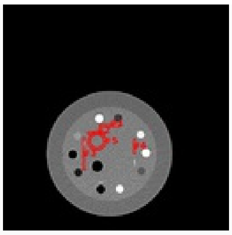

CTP:Uniformity:info_v1

Metal or blank insert data information

Image Number	Target size (small or large)	Max pixel value
63	Large	80

Metal Artefact ROI analysis

Metal Artefact ROI analysis. ROIs are displayed on thumbnail.

ROI#	Mean	SD	Range
ROI #1	47.7	42.6	300
ROI #2	30.5	15.1	79.0
ROI #3	47.2	38.7	247
ROI #4	34.6	17.4	103
ROI #5	33.4	17.3	104

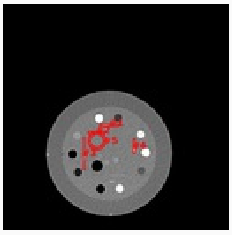

CTP:Uniformity:info_v1

Metal or blank insert data information

Image Number	Target size (small or large)	Max pixel value
66	Large	77

Metal Artefact ROI analysis

Metal Artefact ROI analysis. ROIs are displayed on thumbnail.

ROI#	Mean	SD	Range
ROI #1	45.4	41.3	291
ROI #2	43.4	25.4	161
ROI #3	52.1	40.9	269
ROI #4	61.3	58.5	287
ROI #5	34.1	15.5	127

Warnings

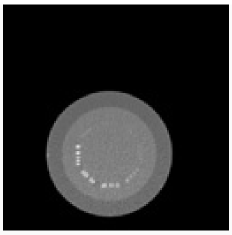

Slices from the CTP714 module were identified. Currently, the service is not reporting information on the high resolution gauges as it is a subjective visual test. Please upload a slice from the CTP682 module including the MTF bead.

File name: CATPHAN.CT.THORAX_THORAX_1_(ADULT).0001.0053.2018.07.11.18.06.26.501374.150367719
Series description: Thorax + C 3.0 I30f 2
Module ID: CTP714
Total collimation width: 1.920000e+01 mm
mA: 151
Time: 500
kVp: 80
Slice thickness: 3.00 mm
Scan mode: N/A

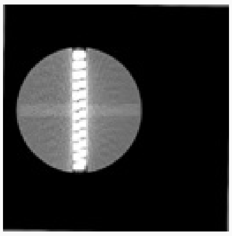

The wave harmonics analysis requires slice thickness to be less than 2mm. Please upload a thinner slice.

support@imageowl.com / Terms of use / Copyright © 2018 Image Owl
